# Comparative Efficacy of Ultrasound‐Guided Quadratus Lumborum Block Versus Alternative Fascial Plane Blocks for Postoperative Analgesia Following Hysterectomy: A Systematic Review and Meta‐Analysis

**DOI:** 10.1155/anrp/5760922

**Published:** 2026-05-17

**Authors:** Priyanka Dwivedi, Tejas K. Patel, Vijeta Bajpai, Chanchal Goyal, Ankita Kabi, Santosh Kumar Sharma, Hari Shanker Joshi, Pratibha Singh

**Affiliations:** ^1^ Department of Anaesthesiology, All India Institute of Medical Sciences, Gorakhpur, Uttar Pradesh, 273008, India, aiims.edu; ^2^ Department of Pharmacology, All India Institute of Medical Sciences, Gorakhpur, Uttar Pradesh, 273008, India, aiims.edu; ^3^ Department of Health Research, Ministry of Health and Family Welfare, New Delhi, India, mohfw.gov.bd; ^4^ Director, ICMR-Regional Medical Research Centre, Gorakhpur, Uttar Pradesh, 273008, India; ^5^ Department of Anaesthesiology, Maharshi Devraha Baba Autonomous State Medical College, Deoria, Uttar Pradesh, 274001, India

**Keywords:** analgesia, hysterectomy, nerve block, pain, postoperative, systematic review

## Abstract

**Objective:**

The primary aim was to compare the efficacy of the quadratus lumborum (QL) block with alternative fascial plane blocks for postoperative analgesia following hysterectomy, while secondary aims were to compare total 24‐h analgesic consumption, postoperative pain scores at different time intervals, and the incidence of postoperative nausea and vomiting (PONV) between the QL block and comparator groups.

**Methods:**

Electronic databases were systematically searched for studies that compared bilateral ultrasound‐guided single‐injection QL block to no block or other fascial plane blocks in hysterectomy surgeries. The QL block was compared with other fascial plane blocks, including transversus abdominis plane (TAP), oblique subcostal TAP (OSTAP), erector spinae plane (ESP), and combined TAP with ilioinguinal/iliohypogastric nerve block. The methodological quality of the included studies was assessed using the RoB 2.0 risk‐of‐bias tool. All outcomes were pooled using the Mantel–Haenszel method and random‐effect model.

**Results:**

After screening 946 relevant articles, 15 RCTs were included in this meta‐analysis. The QL block significantly prolonged the time to first rescue analgesic request compared to the TAP block in the abdominal hysterectomy subgroup (MD 244.77 [95% CI: 191.98 to 297.56], *I* ^2^ = 89%; GRADE evidence—“high”) and the laparoscopic hysterectomy subgroup (MD 443.15 [95% CI: 56.37 to 829.93], *I* ^2^ = 98%; GRADE evidence—“high”). Regarding the 24‐h total analgesic requirement, the QL block significantly reduced its consumption than the TAP block in both the abdominal (MD ‐2.64 [95% CI: −4.19 to −1.09], *I* ^2^ = 93%; GRADE evidence—“moderate”) and the laparoscopic hysterectomy subgroup (MD ‐6.65 [95% CI: −7.39 to −5.91], *I* ^2^ = NA due to *n* = 1; GRADE evidence—“moderate”). The risk of PONV did not differ significantly between the QL block and placebo in abdominal hysterectomy (RR 2.48 [95% CI: 0.50 to −12.35], *I* ^2^ = 0%; GRADE evidence: “moderate”) and laparoscopic hysterectomy arm (RR 0.78 [95% CI: 0.50 to −1.22], *I* ^2^ = 44%; GRADE evidence: “moderate”). The QL block did not show significant difference in risk of PONV than the TAP block across both the abdominal (RR 0.59 [95% CI: 0.25 to 1.40], *I* ^2^ = 0%; GRADE evidence: “moderate”) and laparoscopic hysterectomy subgroups (RR 0.86 [95% CI: 0.30 to 2.45], *I* ^2^ = 0%; GRADE evidence: “moderate”).

**Conclusion:**

The QL block provides superior postoperative analgesia compared with the TAP block in patients undergoing abdominal and laparoscopic hysterectomy.

## 1. Introduction

Hysterectomy is one of the most frequently performed gynecological procedures, utilizing various approaches, including abdominal, vaginal, and minimally invasive methods, such as total laparoscopic or laparoscopically assisted vaginal hysterectomy [1]. Laparoscopic hysterectomies are often preferred due to their benefits of reduced postoperative pain, lower morbidity, faster recovery, and shorter hospital stays [2–4]. Various regional anesthesia techniques, such as intrathecal and epidural opioids, surgical wound infiltration, intraperitoneal local anesthetic instillation, superior hypogastric plexus block [5–7], and fascial plane blocks, have been explored for managing postoperative pain following abdominal or laparoscopic hysterectomy [8–13]. Recent research emphasizes the effectiveness of single‐injection regional blocks, particularly the quadratus lumborum (QL) and erector spinae plane (ESP) blocks, for pain relief after abdominal hysterectomy [14]. The QL block, a relatively novel fascial plane technique, provides effective pain control for both anterior and posterior abdominal walls as it allows broader distribution of local anesthetics through the thoracolumbar fascia (TLF), which extends craniocaudally from the endothoracic fascia to the fascia iliaca [15–17]. It also enables local anesthetic to spread into the paravertebral space, reaching the T10 segment [18], and provides visceral analgesia by targeting the celiac ganglion or sympathetic trunk via splanchnic nerves.

Four variants of the QL block have been identified based on the site of the local anesthetic deposition relative to the QL muscle: QL1 (lateral), QL2 (posterior), QL3 (anterior), and QL4 (intramuscular). These variants offer sensory inhibition from T7 to L1 via TLF spread, making the QL block suitable for major gynecological surgeries [19]. Recent meta‐analyses suggest that the QL block offers superior analgesia for patients undergoing laparoscopic surgeries [20–22]. However, the PROcedure‐SPECific postoperative pain management (PROSPECT) working group did not recommend regional anesthesia techniques for postoperative analgesia after laparoscopic hysterectomy [23].

Several studies compared the QL block to other fascial plane blocks for postoperative analgesia after hysterectomy [11, 24–37], but they have smaller sample sizes and lack of consistency in the results. While prior meta‐analyses have evaluated the QL block in abdominal surgeries broadly or in cesarean delivery, no previous systematic review has simultaneously compared the QL block against multiple alternative fascial plane blocks—including TAP, OSTAP, ESP, and combined TAP with ilioinguinal/iliohypogastric nerve (IIN) block—across both abdominal and laparoscopic hysterectomy within a single, preregistered protocol. The present meta‐analysis addresses this gap by providing a comprehensive, subgroup‐stratified analysis of the QL block’s efficacy, incorporating the randomized controlled trials (RCTs) and applying the GRADE framework to rate the certainty of evidence for each comparison. This approach allows for a more robust and clinically informative synthesis than previously available, directly supporting evidence‐based decision‐making for postoperative analgesic technique selection in hysterectomy. Therefore, this meta‐analysis aims to evaluate the overall effectiveness of the QL block in postoperative pain management for hysterectomy patients compared with other fascial plane blocks, such as transversus abdominis plane (TAP), oblique subcostal TAP (OSTAP), ESP, and combined TAP with IIN blocks.

## 2. Methods

The current systematic review was conducted as per the “Preferred Reporting Items for Systematic Reviews and Meta‐Analyses (PRISMA)” checklist [38]. The study protocol was prospectively registered with PROSPERO: CRD42022322716.

### 2.1. Study Identification

Two investigators (PD and VB) independently and systematically searched the databases (PubMed, PubMed Central, Scopus, LILACS, Google Scholar, trial registry—clinicaltrial.gov, and Cochrane Database of Systematic Reviews) for full‐text articles. The search did not include gray literature (unpublished or preprint studies). A literature search was conducted using the Boolean operator “AND” to combine the search terms: (quadratus lumborum block OR QL block OR quadratus lumborum fascial plane block) AND (hysterectomy) AND (postoperative analgesia). Search: ((“quadratus lumborum block” OR “QL block” OR “QLB” OR “quadratus lumborum fascial plane block”) AND (hysterectomy OR “uterine surgery” OR “abdominal hysterectomy” OR “laparoscopic hysterectomy”)) AND (“postoperative analgesia” OR “postoperative pain” OR “pain management” OR “Pain, Postoperative”[MeSH]). The reference lists of all potentially eligible citations including review articles, systematic reviews, and meta‐analyses were also manually searched to identify additional studies that fulfilled inclusion criteria. The last search was conducted on December 2, 2023. Studies were included regardless of language or publication date, but those in languages other than English were excluded if English translations were unavailable. Initially, two investigators independently assessed titles and abstracts as per the selection criteria. Subsequently, the full texts of relevant studies were assessed to decide the eligibility of retrieved articles. Any disagreements or discrepancies were resolved by discussion and consensus among the authors.

### 2.2. Selection Criteria

The population, intervention, comparison, outcome (PICO) for the meta‐analysis was defined as (P): adult females undergoing abdominal or laparoscopic hysterectomy under spinal or general anesthesia, (I): ultrasound‐guided bilateral QL block for postoperative pain management, regardless of approach, local anesthetic drug, volume, or concentration, (C): no block, placebo, or other fascial plane blocks or abdominal wall blocks, (O): efficacy of the intervention in postoperative pain management (time to the first rescue analgesic after surgery, total analgesic consumption within 24 h, and postoperative pain scores [0–10] at various time intervals of 0–2, 2–6, 6–12, and 12–24 h).

### 2.3. Inclusion Criteria

RCTs compare the efficacy of ultrasound‐guided bilateral QL block with no block or placebo or other fascial plane blocks or abdominal wall blocks in patients undergoing abdominal or laparoscopic hysterectomy.

### 2.4. Exclusion Criteria

• Studies performing the QL block by techniques other than ultrasound guidance
• Observational studies
• Single‐arm studies, case reports, case series, abstract from conference presentations, and review articles.

### 2.5. Efficacy Outcomes

• The primary outcome was the time to the first rescue analgesic after surgery (in minutes). Secondary outcomes included total analgesic consumption within 24 h and postoperative pain scores (0–10) at various time intervals of 0–2, 2–6, 6–12, and 12–24 h.

### 2.6. Safety Outcome

• The number of patients experiencing adverse events (nausea and/or vomiting) in study groups was presented as safety outcomes.

### 2.7. Risk‐of‐Bias Assessment of Included Studies

The ROB‐II tool was used to assess the risk of bias in the included RCTs. Each trial was evaluated by two investigators (PD and TKP) for the predefined set of bias domains that include randomization procedure, deviations from planned interventions, incomplete outcome data, outcome measurement, and selective result reporting. Based on the risk‐of‐bias assessment, the studies were categorized as “low risk,” “high risk,” or having “some concerns” [39]. Both investigators independently assessed a subset of studies and then compared results to discuss and resolve any discrepancies.

### 2.8. Data Extraction

Two reviewers (PD and VB) independently searched for all the clinical trials using retrieved titles and abstracts from the databases. The following data were extracted: first author, publication year, study design, demographics, anesthesia modality used, type of hysterectomy, technique of the QL block (anterior, posterior, or lateral), timing of block (pre‐anesthesia, preoperative, or postoperative), local anesthetics and adjuvants used (type, dose, and volume), comparator technique, number of participants in each treatment arm, follow‐up duration, time to first analgesic requirement after surgery, analgesic consumption in 24 h of surgery, analgesic efficacy, and safety outcomes. Data reported as median (range and/or interquartile range) were converted into mean (standard deviation) using an online tool [40] based on Luo et al. [41] and Wan et al. [42]. Similarly, numerical data for outcomes presented as graphs were extracted for quantitative analysis using the standardized software WebPlotDigitizer [43]. Additionally, all extractions were performed independently by two reviewers, followed by a cross‐verification process to resolve any discrepancies. This approach ensured a robust and consistent methodology for incorporating visual data into our quantitative analysis. For postoperative analgesic consumption during the first 24 h, data were presented as oral/intravenous morphine, pethidine, tramadol, sufentanil, and cumulative analgesic consumption score (CACS) across the different trials. All drugs were converted to intravenous morphine milligram equivalents (MME) for analysis (Supporting table 1) [44–47]. Pain scores were reported in the form of visual analog scale (0–10, 0–100), numerical rating scale (0–10), and verbal rating scale (0–10). For the standardization of the data, all the pain scores were converted on the uniform scale of 0–10. Pain scores were reported at different time points or intervals across studies. To assess the effect of QL block intervention, we used data from time intervals (0–2, 2–6, 6–12, and 12–24 h) rather than specific points. For quantitative analysis, later time points within an interval were included (e.g., for data at 30 min, 1 h, and 2 h, we used the 2‐h data for the “0–2 h” interval postoperatively). These conversions were cross‐checked for consistency across multiple reviewers (PD, TKP, VB, and AK). The data were collected in an Excel sheet and verified by another investigator (TKP) to ensure accuracy. Trials being excluded were reviewed by both authors (PD and VB) before the final agreement. Any disagreements regarding study selection and exclusion were resolved by discussion and consensus among the investigators or by a third investigator (TKP/AK) in the meta‐analysis.

### 2.9. Data Synthesis

The effect sizes were summarized as a mean difference (MD) or standardized mean difference (SMD) with 95% confidence interval (CI) in the case of continuous data and as a risk ratio (RR) with 95% CI in the case of dichotomous data (adverse events). The pooled meta‐analytic summaries were estimated through the Mantel–Haenszel method using a random‐effect model with the DerSimonian–Laird approach. The heterogeneity was assessed using the *I* ^2^ test. An *I* ^2^ > 50% was used to consider significant statistical heterogeneity in the meta‐analytic summary estimate. A forest plot was used for the graphical display of the results of individual studies and meta‐analytic summaries of each outcome.

Several studies included multiple arms, comparing the QL block with placebo as well as other fascial plane blocks. Data from each control arm were incorporated into the subgroup analysis. However, to avoid overestimating the results due to potential duplication of participants or data, a total pooled analysis was not conducted.

Based on the findings of the preliminary search and extracted data, we anticipated the two surgical groups (laparoscopic hysterectomy and abdominal hysterectomy) and many comparator groups of fascial plane blocks (placebo, TAP block, combined TAP with iliohypogastric‐IIN block, OSTAP block, and ESP block). Subgroup analysis of primary and secondary outcomes was conducted based on the number of studies, categorizing by surgery type (laparoscopic or abdominal hysterectomy), and control group (placebo or other fascial plane blocks). The risk‐of‐bias domain was assessed based on the proportion of information derived from studies with high or unclear risk of bias, the percentage of weight carried by these studies in the meta‐analysis, and the impact of excluding these studies through sensitivity analysis on the meta‐analytic summary.

The sensitivity analyses of each outcome were performed based on the risk‐of‐bias assessment as per the ROB‐II tool for RCTs. Studies identified with “some concern” or “high” risk of bias were excluded to generate the meta‐analytic summary for each outcome [39].

Meta‐regression was conducted to investigate potential sources of heterogeneity in the primary outcome variable based on the following study characteristics: comparator types (placebo, TAP block, TAP with IIN block, and OSTAP block), types of surgery (abdominal or laparoscopic hysterectomy), anesthesia methods (general or local), and drugs used in peripheral blocks (bupivacaine, ropivacaine, or bupivacaine with dexmedetomidine). Initially, univariable meta‐regression was used to evaluate the impact of these characteristics on primary efficacy outcomes. Variables with a significance level of *p* < 0.10 were further analyzed using multivariable meta‐regression, and those with a *p*‐value < 0.05 were identified as statistically significant predictors of heterogeneity in the model.

### 2.10. Certainty of Evidence

The certainty of the evidence was rated for each outcome using the predefined set of domains of the GRADE approach. The domains included risk of bias, inconsistency, imprecision, indirectness, and publication bias. For inconsistency, evidence was rated based on the degree of heterogeneity (*I* ^2^ > 50%) and the direction of effect sizes among the included studies for each outcome. For imprecision, studies were classified as imprecise if the CIs for the effect estimates were wide and did not meet the prespecified criteria for superiority, inferiority, or equivalence (Table 1). For example, we applied a threshold of greater than or less than 60 min for superiority or inferiority, and ±60 min for equivalence, to classify time to first rescue analgesia outcomes as imprecise. For indirectness, evidence was considered indirect if the population, intervention, or outcome of the study differed significantly from the review’s focus. The PICO framework was used to evaluate the level of indirectness. Publication bias was assessed through visual inspection of funnel plots and Egger’s regression test. A significant intercept (*p* < 0.05) in Egger’s regression test was considered indicative of publication bias. Evidence was downgraded by one level (from high to moderate) for a serious concern in any single domain, by two levels (from high to low) for serious concerns in two domains, and by three levels (from high to very low) for serious concerns in three or more domains. The quality of evidence for each outcome was rated as high, moderate, low, or very low. The GRADEpro software was used to construct the summary of findings table [48, 49]. To ensure consistency in applying the GRADE framework, both investigators used the official GRADE handbook. Additionally, we followed standardized protocols outlined in the GRADE approach, including detailed discussions among investigators to resolve uncertainties and ensure uniform application of the criteria across all assessments. The meta‐analysis was conducted through the “Review Manager software version 5.4.1” and meta‐regression through the “JASP software version 0.19.2.0.”

**TABLE 1:** Interpretation of efficacy and safety outcomes.

Outcome	Effect size	Superiority	Comparable or equivalent range
Time for first rescue analgesia	Mean difference	> 60 min	−60–60 min
Analgesic consumption (morphine equivalent) 24 h	Mean difference	> 10 mg	−10–10 mg
Pain scores at various time intervals (0–2 h, 2–6 h, 6–12 h, and 12–24 h)	Standardized mean difference	> 20 units	−0.20–0.20 unit
Postoperative nausea and/or vomiting	Risk ratio	< 0.8	0.80–1.20

## 3. Results

### 3.1. Study Characteristics

**FIGURE 1:**
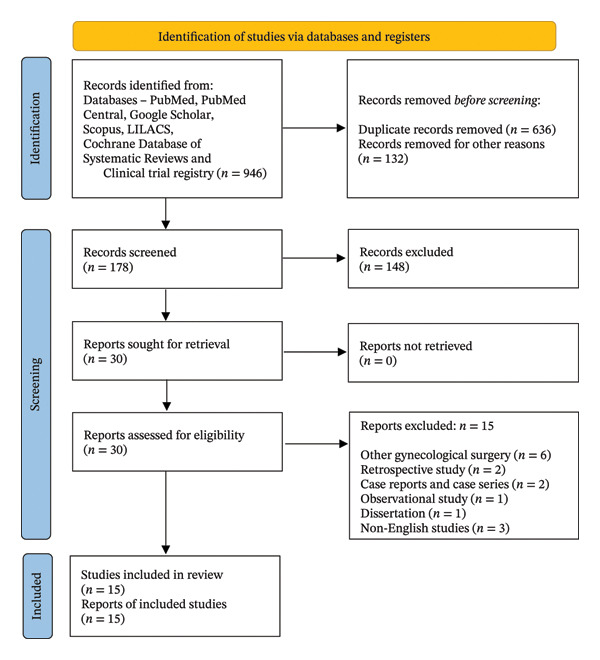
PRISMA flow diagram showing the study selection process. Source: Page MJ, et al. BMJ 2021; 372: n71. doi: 10.1136/bmj.n71. This work is licensed under CC BY 4.0. To view a copy of this license, visit https://creativecommons.org/licenses/by/4.0/.

From the literature search, we retrieved 946 references and assessed 30 full‐text articles. A total of 15 RCTs with 1130 patients fulfilling the selection criteria were included in the analysis (Figure 1) [11, 24–37].

The surgical procedure was laparoscopic in eight studies (11, 19, 21–25, and 28) and abdominal in seven studies (18, 20, 26–27, and 29–31). Among the included studies, seven studies compared bilateral QL block with no block or placebo (11, 19, 21, 23, 26, 28, and 29), eight studies compared with bilateral TAP block (18, 20, 24–26, and 29–31), two studies compared with the ESP block (19 and 23), and one study each compared with bilateral coupled block with TAP and IIN block [27] and OSTAP block [22]. Among 15 included studies, the technique of the QL block was lateral in one study (20), posterior in five studies (24–26, 28, and 31), anterior (transmuscular) in seven studies (11, 19, 21–23, 27, and 29), and combined posterior and anterior in one study (30). One study did not specify the technique of the QL block (18). The general characteristics of the included studies are summarized in Table 2. The number of patients, demographic characteristics, and duration of surgery between intervention and comparative arms are summarized in Table 3.

**TABLE 2:** General characteristics of included studies.

Study	Study design	Type of blinding	*N*	Drug for peripheral block	Drug concentration (%)	Drug volume (mL)	Type of anesthesia	Type of surgery	Block timing	Pain rating scale	Rescue analgesia	Outcomes
Alansary et al. (2022) [29]	RCT	Open	60	NS	NS	NS	GA	TAH	After GA induction	VAS (0–10)	IV pethidine 50 mg	VAS scores at 2, 4, 6, 12, 18, and 24 h, time for 1st rescue analgesic, 24‐h pethidine used, and PONV
Baran et al. (2023) [30]	RCT	Open	81	Bupivacaine	0.25	30	GA	TAH	Before GA induction	VAS (0–10)	IV pethidine 50 mg	VAS scores at 2, 6, 12, and 24 h, time for 1st rescue analgesic, 24‐h opioid consumption, and PONV
Dixit et al. (2021) [31]	RCT	Double	70	Bupivacaine	0.25	20	SA	TAH	After surgery	NPIS (0–10)	Morphine	NPIS at 2, 4, 6, 8, 12, 16, and 24 h, time for 1st rescue analgesic, and total analgesic consumption
Hansen et al. (2020) [32]	RCT	Double	70	Ropivacaine	0.375	30	GA	TLH	Before GA induction	NRS (0–10)	IVPCA with morphine	NRS at 6, 12, 24, and 24 h, cumulative analgesic consumption, time to 1st rescue analgesic, and PONV
Huang et al. (2021) [33]	RCT	Open	60	Ropivacaine	0.375	20	GA	TLH	Before GA induction	NRS (0–10)	IVPCA with morphine	NRS at 30 min, 1, 2, 6, 12, 18, 24, and 24 h, cumulative morphine consumption, time to 1st rescue analgesic, and PONV
Jadon et al. (2021) [22]	RCT	Double	74	Ropivacaine	0.375	20	GA	TLH	After GA induction	VAS (0–10)	IVPCA with fentanyl	VAS scores at 2, 6, 12, and 24 h, time for 1st rescue analgesic, 24‐h opioid consumption, and PONV
Jiang et al. (2023) [34]	RCT	Open	90	Ropivacaine	0.4	25	GA	TLH	Before GA induction	NRS (0–10)	IV pentazocine 30 mg	24‐h sufentanil consumption and PONV
Kothapalli et al. (2020) [35]	RCT	Open	60	Bupivacaine and dexmedetomidine	0.25 and 20 mcg	20	GA	TLH	After surgery	VAS (0–10)	IV paracetamol 1 gm	VAS at 30 min, 2, 4, 8, 16, and 24 h, time to 1st rescue analgesic, and PONV
Midathala et al. (2022) [36]	RCT	Open	140	Bupivacaine and dexmedetomidine	0.25 + 20 mcg	20	GA	TLH	After surgery	VAS (0–10)	IV tramadol 50 mg followed by 25 mg every 15 min	VAS at 1, 2, 4, 8, 10, 12, 14, 16, 18, 20, 22, and 24 h, time to 1st rescue analgesic, 24‐h tramadol consumption, and PONV
Naaz et al. (2021) [37]	RCT	Double	76	Bupivacaine	0.25	20	GA	TAH	After surgery	VRS (0–10)	IV diclofenac, IV tramadol, and IV morphine	VRS at cumulative analgesic consumption, time to 1st rescue analgesia, and PONV
Omara et al. (2023) [38]	RCT	Open	60	Bupivacaine	0.25	20	SA	TAH	After spinal anesthesia	VAS (0–10)	IV morphine 3 mg and paracetamol 1 mg	VAS at 30 min, 2, 4, 6, 12, and 24 h, time to 1st rescue analgesic, 24‐h analgesic consumption, and PONV
Peng et al. (2022) [39]	RCT	Double	60	Ropivacaine	0.375	22	GA	TLH	After GA induction	VAS (0–10)	Flurbiprofen axetil	VAS scores at 2, 6, 12, 24, and 24 h, 24‐h opioid consumption, and PONV
Shukla et al. (2021) [40]	RCT	Double	105	Bupivacaine	0.25	20	SA	TAH	After surgery	VAS (0–10)	IM diclofenac, IV tramadol, and IV morphine	VAS at 15 min, 30 min, 1, 2, 6, 12, and 24 h, time to 1st rescue analgesic, and PONV
Shukla et al. (2023) [41]	RCT	Double	70	Bupivacaine	0.25	20	SA	TAH	After surgery	NRS (0–10)	IM diclofenac and IV tramadol	Time to 1st rescue analgesic and PONV
Yousef et al. (2018) [42]	RCT	Double	60	Bupivacaine	0.25	20	GA	TAH	After GA induction	VAS (0–10)	IV increment of morphine 3 mg	VAS at 30 min, 2, 4, 6, 12, and 24 h, time to 1st rescue analgesic, 24‐h morphine consumption, and PONV

*Note: N*, total number of patients; IVPCA, intravenous patient‐controlled analgesia; PONV, postoperative nausea and vomiting.

Abbreviations: GA, general anesthesia; NPIS, numerical pain intensity scale; NRS, numerical rating scale; NS, not specified; RCT, randomized controlled trial; SA, spinal anesthesia; TAH, total abdominal hysterectomy; TLH, total laparoscopic hysterectomy; VAS, visual analog scale; VRS, verbal rating scale.

**TABLE 3:** Characteristics of intervention and comparative arms.

Study	Type of block		No of patients		Age mean (SD)		Height mean (SD)		Weight mean (SD)		Duration of surgery (min) mean (SD)	
	QLB	Comparator block	QLB	Comparator	QLB	Comparator block	QLB	Comparator block	QLB	Comparator block	QLB	Comparator block
Alansary et al. (2022) [29]	NS	Bilateral TAP	30	30	52.1 (6.3)	54.3 (3.6)	169.8 (6.96)	160.2 (33.9)	82.5 (11.6)	81.1 (11.8)	149.67 (54.74)	145.33 (45.62)
Baran et al. (2023) [30]	TQLB	No block and bilateral ESPB	27	27 and 27	48.5 (8.3)	49.6 (10.0) and 50.9 (6.2)	160 (10)	160 (10) and 160 (10)	70.3 (8.5)	69.5 (8.8) and 71.6 (7.7)	156.1 (61.5)	153.9 (28.2) and 168.1 (37.6)
Dixit et al. (2021) [31]	Lateral QLB	Bilateral TAP	35	35	39.20 (11.70)	38.34 (11.64)	150.53 (6.23)	152 (6.26)	52.57 (9.65)	52.3 (6.73)	NS	NS
Hansen et al. (2020) [32]	TQLB	Sham block	30	30	62.2 (11.7)	63.4 (10.9)	NS	NS	80.4 (16.9)	80.2 (17.5)	116.1 (61.7)	122.5 (66.1)
Huang et al. (2021) [33]	TQLB	Bilateral OSTAP	30	30	52.6 (7.5)	50.6 (6.2)	158.8 (5.2)	158.5 (5.6)	59.8 (8.4)	59.2 (8.9)	NS	NS
Jadon et al. (2021) [22]	TQLB	Bilateral sham block	37	37	43.5 (7.9)	43.5 (4.4)	150.8 (4.9)	151.6 (5.7)	61.3 (7.5)	63.2 (8.2)	153 (38)	150 (42)
Jiang et al. (2023) [34]	TQLB	No block and bilateral ESPB	30	30 and 30	50.8 (5.6)	50.7 (5.9) and 51.5 (4.6)	NS	NS	NS	NS	102.3 (39.0)/	97.4 (36.6) and 100.4 (29.1)
Kothapalli et al. (2020) [35]	Posterior QLB	Bilateral TAP block	30	30	56.5 (6.97)	50.70 (6.8)	NS	NS	71.23 (7.22)	72.23 (6.37)	107 (40)	122 (42)
Midathala et al. (2022) [36]	Posterior QLB	Bilateral TAP block	70	70	49.52 (3.85)	49.6 (3.96)	159.15 (3.71)	159.62 (4.11)	58.2 (3.71)	58.62 (4.706)	101.67 (7.71)	101.54 (8.207)
Naaz et al. (2021) [37]	Posterior QLB	No block and bilateral TAP block	25	26 and 26	43.6 (8.54)	42.52 (8.27) and 43.96 (6.44)	NS	NS	NS	NS	NS	NS
Omara et al. (2023) [38]	TQLB	Bilateral coupled block (TAP and IIN block)	30	30	53.8 (5.1)	53 (6.2)	NS	NS	NS	NS	107.1 (5.1)	106.8 (9.8)
Peng et al. (2022) [39]	Posterior QLB	Bilateral sham block	30	30	47.50 (5.64)	46.90 (6.02)	161.57 (3.16)	160.53 (2.80)	62.02 (6.72)	62.12 (7.81)	84.03 (26.03)	75.40 (20.70)
Shukla et al. (2021) [40]	QLB type NS	No block and bilateral TAP block	35	35 and 35	42.54 (5.11)	41.69 (7.52) and 42.8 (5.83)	NS	NS	NS	NS	104.43 (17.05)	103.71 (16.05)) and 103.14 (15.43)
Shukla et al. (2023) [41]	Combined posterior + anterior QLB	Bilateral TAP block	35	35	46.69 (8.23)	46.37 (7.82)	NS	NS	NS	NS	104.71 (21.11)	98.43 (18.1)
Yousef et al. (2018) [42]	Posterior QLB	Bilateral TAP block	30	30	56.5 (6.97)	50.70 (6.8)	NS	NS	71.23 (7.22)	72.23 (6.37)	107 (40)	122 (42)

Abbreviations: ESPB, erector spinae plane block; NS, not specified; OSTAP, oblique subcostal TAP; QLB, quadratus lumborum block; SD, standard deviation; TAP, transverse abdominis plane.

### 3.2. Risk of Bias in Included Studies

The risk‐of‐bias assessment in individual RCTs is presented in Supporting Figure 1. In the overall risk‐of‐bias assessment, six studies were considered to have “some concern” as per the ROB‐II tool [39], and nine RCTs were considered to have a “low” risk of bias.

### 3.3. Primary Efficacy Outcomes

#### 3.3.1. Time to First Rescue Analgesia

**FIGURE 2:**
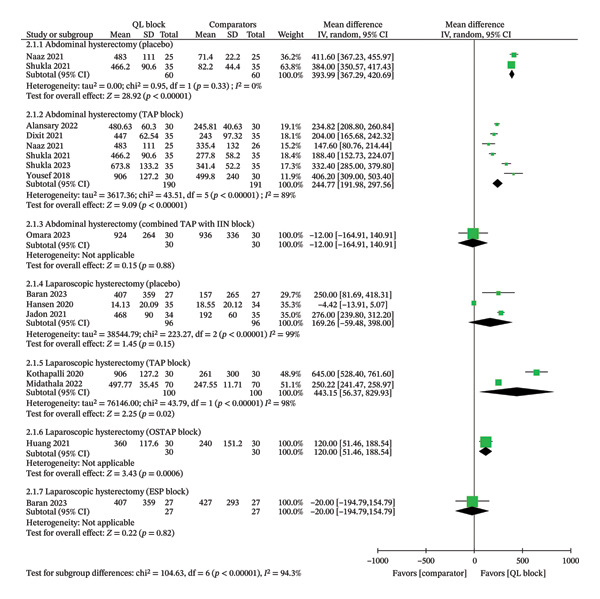
Meta‐analytic summary of the time to first rescue analgesia through a random‐effect model. QL block—quadratus lumborum block; TAP block—transversus abdominis plane block; IIN block—ilioinguinal nerve block; OSTAP block—oblique subcostal transversus abdominis plane block; ESP block—erector spinae plane block; SD—standard deviation; CI—confidence interval; IV—inverse variance.

Thirteen trials involving 980 patients reported this outcome as the time when the first rescue analgesic was given after surgery. The outcome was presented in either hours or minutes among the included trials. All data were converted into minutes. In the abdominal hysterectomy subgroup, the QL block showed superiority over placebo (MD 393.99; 95% CI, 367.29, 420.69, *I* ^2^ = 0%; GRADE evidence: high) and the TAP block (MD 244.77; 95% CI, 191.98, to 297.56, *I* ^2^ = 89%; GRADE evidence: high) and a trend of equivalence with the combined TAP with the IIN block (MD ‐12.00; 95% CI, −164.91, to 140.91, *I* ^2^ = NA; GRADE evidence: low). In the laparoscopic hysterectomy subgroup, the QL block showed a trend of superiority over TAP (MD 443.15; 95% CI, 56.37, to 829.93, *I* ^2^ = 98%; GRADE evidence: high) and OSTAP block (MD 120.00; 95% CI, 51.46 to 188.54, *I* ^2^ = NA; GRADE evidence: moderate); however, no significant difference was present compared to placebo (MD 169.26; 95% CI, −59.48, to 398.00, *I* ^2^ = 99%; GRADE evidence: low) and ESP block (MD ‐20.00; 95% CI, −194.79, to 154.79, *I* ^2^ = NA; GRADE evidence: moderate) (Figure 2).

No significant asymmetry in the funnel plot was observed (Egger’s regression test for funnel plot asymmetry: *Z* score = 0.428, *P* = 0.669) (Supporting Data File—Supporting Figure 2a). A sensitivity analysis based on the risk‐of‐bias assessment did not affect the overall findings (Supporting Data file—Supporting Table 2). The meta‐regression did not identify any significant predictors for the time to first rescue analgesia outcome across comparator types, types of surgery, anesthesia methods, or drugs used in peripheral blocks (Supporting Table 3).

### 3.4. Secondary Efficacy Outcomes

#### 3.4.1. Total Analgesic Consumption in 24 h

Twelve trials (*n* = 895) reported this outcome. The conversion of total analgesic consumption in MME in each study is presented in Supporting table 4. The analysis observed a significantly lower analgesic consumption in 24 h in patients receiving the QL block for postoperative analgesia when compared to placebo (MD‐1.94; 95% CI, −2.26 to −1.62 *I* ^2^ = NA; GRADE evidence: low) or TAP block (MD‐2.64; 95% CI, −4.19 to −1.09 *I* ^2^ = 93%; GRADE evidence: moderate), and no significant difference was found compared to the combined TAP with the IIN block (MD ‐0.10; 95% CI, −0.97 to 1.17, *I* ^2^ = NA; GRADE evidence: low) for abdominal hysterectomy patients. Among the laparoscopic hysterectomy subgroup, patients receiving the QL block had lower analgesic consumption when compared to placebo (MD ‐3.82; 95% CI, −5.77 to −1.88, *I* ^2^ = 47%; GRADE evidence: moderate), TAP block (MD ‐6.65; 95% CI, −7.39 to −5.91, *I* ^2^ = NA; GRADE evidence: moderate), OSTAP block (MD ‐8.90; 95% CI, −15.43 to −2.37, *I* ^2^ = NA; GRADE evidence: moderate), and equivalent to the ESP block (MD ‐0.34; 95% CI, −1.65 to 2.33, *I* ^2^ = 0%; GRADE evidence: moderate) (Figure - 3). The GRADE approach suggested a “low to moderate quality” of evidence (Table - 4). A significant asymmetry in the funnel plot was observed (Egger’s regression test for funnel plot asymmetry: *Z* score = −2.274, *P* = 0.023) (Supporting Data File—Supporting Figure 2b). A sensitivity analysis based on the risk‐of‐bias assessment did not affect the overall findings (Supporting Data file—Supporting Table 2).

**FIGURE 3:**
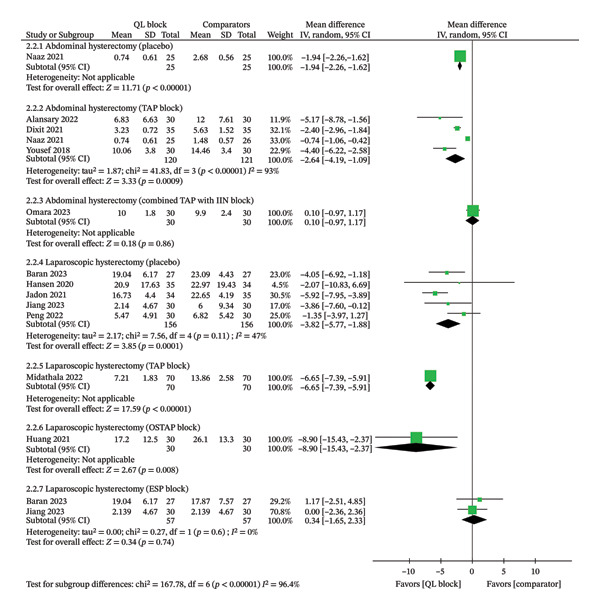
Meta‐analytic summary of the analgesic consumption through a random‐effect model. QL block—quadratus lumborum block; TAP block—transversus abdominis plane block; IIN block—ilioinguinal nerve block; OSTAP block—oblique subcostal transversus abdominis plane block; ESP block—erector spinae plane block; SD—standard deviation; CI—confidence interval; IV—inverse variance.

**TABLE 4:** GRADE quality of evidence for the time for 1^st^ rescue analgesia, analgesic consumption in 24 h, and postoperative nausea and vomiting outcomes.

Outcomes	No of studies	Study design	Risk of bias	Inconsistency	Indirectness	Imprecision	Other considerations	Effect estimate MD (95% CI)/SMD (95% CI)^∗^/RR (95% CI)^∗∗^	Certainty
*Time for 1st rescue analgesia*									
Abdominal hysterectomy (placebo)	2	RCTs	Not serious	Not estimable	Not serious	Not serious	Publication bias undetected	393.99 [367.29, 420.69]	⨁⨁⨁⨁ High
Abdominal hysterectomy (TAP block)	6	RCTs	Not serious	Not serious	Not serious	Not serious	Publication bias undetected	244.77 [191.98, 297.56]	⨁⨁⨁⨁ High
Abdominal hysterectomy (combined TAP with IIN block)	1	RCT	Serious^a^	Not estimable	Not serious	Serious^c^	Publication bias undetected	−12.00 [‐164.91, 140.91]	⨁⨁◯◯ Low
Laparoscopic hysterectomy (placebo)	3	RCTs	Not serious	Serious^b^	Not serious	Serious^c^	Publication bias undetected	169.26 [‐59.48, 398.00]	⨁⨁◯◯ Low
Laparoscopic hysterectomy (TAP block)	2	RCTs	Not serious	Not serious	Not serious	Not serious	Publication bias undetected	443.15 [56.37, 829.93]	⨁⨁⨁⨁ High
Laparoscopic hysterectomy (OSTAP block)	1	RCT	Not serious	Not serious	Not serious	Serious	Publication bias undetected	120.00 [51.46, 188.54]	⨁⨁⨁◯ Moderate
Laparoscopic hysterectomy (ESP block)	1	RCT	Not serious	Not serious	Not serious	Serious	Publication bias undetected	−20.00 [‐194.79, 154.79]	⨁⨁⨁◯ Moderate

*Analgesic consumption in 24 h*									
Abdominal hysterectomy (placebo)	1	RCT	Not serious	Not serious	Not serious	Not serious	Publication bias strongly suspected^a^	−1.94 [‐2.26, −1.62]	⨁⨁◯◯ Low
Abdominal hysterectomy (TAP block)	4	RCTs	Not serious	Not serious	Not serious	Not serious	Publication bias strongly suspected^a^	−2.64 [‐4.19, −1.09]	⨁⨁⨁◯ Moderate
Abdominal hysterectomy (combined TAP with IIN block)	1	RCT	Serious^a^	Not serious	Not serious	Not serious	Publication bias strongly suspected^a^	0.10 [‐0.97, 1.17]	⨁⨁◯◯ Low
Laparoscopic hysterectomy (placebo)	5	RCTs	Not serious	Not serious	Not serious	Not serious	Publication bias strongly suspected^a^	−3.82 [‐5.77, −1.88]	⨁⨁⨁◯ Moderate
Laparoscopic hysterectomy (TAP block)	1	RCT	Not serious	Not serious	Not serious	Not serious	Publication bias strongly suspected^a^	−6.65 [‐7.39, −5.91]	⨁⨁⨁◯ Moderate
Laparoscopic hysterectomy (OSTAP block)	1	RCT	Not serious	Not serious	Not serious	Not serious	Publication bias strongly suspected^a^	−8.90 [‐15.43, −2.37]	⨁⨁⨁◯ Moderate
Laparoscopic hysterectomy (ESP block)	2	RCTs	Not serious	Not serious	Not serious	Not serious	Publication bias strongly suspected^a^	0.34 [‐1.65, 2.33]	⨁⨁⨁◯ Moderate

*Postoperative nausea and vomiting*									
Abdominal hysterectomy (placebo)	2	RCTs	Not serious	Not serious	Not serious	Serious^c^	Publication bias undetected	2.48 [0.50, 12.35]^∗∗^	⨁⨁⨁◯ Moderate
Abdominal hysterectomy (TAP block)	5	RCTs	Not serious	Not serious	Not serious	Serious^c^	Publication bias undetected	0.59 [0.25, 1.40]^∗∗^	⨁⨁⨁◯ Moderate
Abdominal hysterectomy (combined TAP with IIN block)	1	RCT	Serious^a^	Not serious	Not serious	Serious^c^	Publication bias undetected	0.80 [0.24, 2.69]^∗∗^	⨁⨁◯◯ Low
Laparoscopic hysterectomy (placebo)	4	RCTs	Not serious	Not serious	Not serious	Serious^c^	Publication bias undetected	0.78 [0.50, 1.22]^∗∗^	⨁⨁⨁◯ Moderate
Laparoscopic hysterectomy (TAP block)	2	RCTs	Not serious	Not serious	Not serious	Serious^c^	Publication bias undetected	0.86 [0.30, 2.45]^∗∗^	⨁⨁⨁◯ Moderate
Laparoscopic hysterectomy (OSTAP block)	1	RCT	Not serious	Not serious	Not serious	Serious^c^	Publication bias undetected	0.50 [0.24, 1.06]^∗∗^	⨁⨁⨁◯ Moderate
Laparoscopic hysterectomy (ESP block)	1	RCT	Not serious	Not serious	Not serious	Serious^c^	Publication bias undetected	1.18 [0.55, 2.56]^∗∗^	⨁⨁⨁◯ Moderate

*Note:* OSTAP, oblique subcostal transversus abdominis plane block.

Abbreviations: CI, confidence interval; IIN, ilioinguinal and iliohypogastric nerves; MD, mean difference; SMD, standardized mean difference; RCTs, randomized controlled trials; RR, risk ratio; TAP, transabdominal plane.

^∗^Indicates SMD (95% CI).

^∗∗^Indicates RR (95% CI).

^a^Asymmetrical funnel plot.

^b^Evidence based on studies showing “some concern” on risk‐of‐bias assessment.

^c^Wide confidence interval; d. high heterogeneity.

#### 3.4.2. Pain Score at 0–2 h After Interventions

A total of 11 studies (*n* = 825 patients) reported a “pain score at 0–2 h interval postoperatively.” We found a significantly lower pain score in the QL block group when compared to placebo (SMD ‐4.65; 95% CI, −5.47 to −3.65, *I* ^2^ = NA) or TAP block (SMD ‐1.39; 95% CI, −2.30 to −0.47, *I* ^2^ = 91%), and no difference was found compared to the combined TAP with the IIN block (SMD ‐0.00; 95% CI, −0.51 to 0.51, *I* ^2^ = NA) for abdominal hysterectomy patients (Supporting Data File—Supporting Figure 3a). In the laparoscopic hysterectomy subgroup, the QL block showed superiority over placebo (SMD ‐0.41; 95% CI, −0.73 to −0.09, *I* ^2^ = 15%) and OSTAP block (SMD ‐0.59; 95% CI, −1.07 to −0.11, *I* ^2^ = NA); however, no significant difference in pain score was found compared to the TAP block (SMD ‐1.48; 95% CI, −3.33 to 0.36, *I* ^2^ = 96%) or ESP block (SMD ‐0.14; 95% CI, −0.40 to 0.67, *I* ^2^ = NA). A significant asymmetry in the funnel plot was observed (Egger’s regression test for funnel plot asymmetry: *Z* score = −5.317, *p* < 0.001) (Supporting Data File—Supporting Figure 3b). A sensitivity analysis based on the risk‐of‐bias assessment did not affect the overall findings (Supporting Data file—Supporting Table 2).

#### 3.4.3. Pain Score at 2–6 h After Intervention

This outcome was reported in 12 studies (*n* = 894). The overall analysis of this outcome revealed significantly lower pain scores in the QL block group when compared to other fascial plane blocks (SMD ‐2.18; 95% CI, −3.38 to −0.97, *I* ^2^ = 97%). The analysis of the abdominal hysterectomy subgroup showed that the QL block is superior to the placebo (SMD ‐2.36; 95% CI, −2.98 to −1.74, *I* ^2^ = NA) and TAP block (SMD ‐1.66; 95% CI, −2.40 to −0.91, *I* ^2^ = 85%) (Supporting Data File—Supporting Figure 4a). However, the QL block was neither superior nor equivalent to the combined TAP with the IIN block (SMD 0.00; 95% CI, −0.51 to 0.51, *I* ^2^ = NA). Also, in the laparoscopic hysterectomy subgroup, the QL block showed superiority over placebo (SMD ‐0.47; 95% CI, −0.82 to −0.12, *I* ^2^ = 48%) and TAP block (SMD ‐5.24; 95% CI, −6.60 to −3.89, *I* ^2^ = 79%), and no significant difference was found over OSTAP block (SMD ‐0.46; 95% CI, −0.94 to 0.01, *I* ^2^ = NA) and ESP block (SMD 0.13; 95% CI, −0.40 to 0.66, *I* ^2^ = NA) (Table 3). A significant asymmetry in the funnel plot was observed (Egger’s regression test for funnel plot asymmetry: *Z* score = −6.718, *p* < 0.001) (Supporting Data File—Supporting Figure 4b). A sensitivity analysis based on the risk‐of‐bias assessment did not affect the overall findings (Supporting Data file—Supporting Table 2).

#### 3.4.4. Pain Score at 6–12 h After Intervention

Twelve studies including 894 patients presented data for the “pain score at 6–12 h” time interval. In the abdominal hysterectomy subgroup, when compared to the placebo group, the QL block group showed a significantly lower pain scores (SMD ‐0.76; 95% CI, −1.25 to −0.28, *I* ^2^ = NA), but no difference was found compared to the TAP block (SMD ‐1.08; 95% CI, −2.29 to 0.13, *I* ^2^ = 95%) and combined TAP with the IIN block (SMD 0.00; 95% CI, −0.51 to −0.51, *I* ^2^ = NA) (Supporting Data File—Supporting Figure 5a). In the laparoscopic hysterectomy subgroup, pain scores were significantly lower at this time interval in the QL block group when compared to the TAP block (SMD ‐3.39; 95% CI, −4.55, to −2.23, *I* ^2^ = 81%) and OSTAP block (SMD ‐0.70; 95% CI, −1.19, to −0.22, *I* ^2^ = NA); no significant difference was found for placebo (SMD ‐0.39; 95% CI, −1.09, to 0.32, *I* ^2^ = 87%) and ESP block (SMD ‐0.08 95% CI, −0.45, to 0.61, *I* ^2^ = NA). A significant asymmetry in the funnel plot was observed (Egger’s regression test for funnel plot asymmetry: *Z* score = −3.930, *p* < 0.001) (Supporting Data File—Supporting Figure 5a). A sensitivity analysis based on the risk‐of‐bias assessment did not affect the overall findings (Supporting Data file—Supporting Table 2).

#### 3.4.5. Pain Score at 12–24 h After Intervention

Pain scores from 12 h to 24 h were reported in 12 studies (*n* = 894). In the abdominal hysterectomy subgroup, pain scores were lower in QL block group patients compared to placebo only (SMD ‐0.90; 95% CI, −1.39 to −0.40, *I* ^2^ = NA), and no significant difference was observed for the TAP block (SMD ‐1.02; 95% CI, −2.17 to 0.13, *I* ^2^ = 94%) and combined TAP with the IIN block (SMD 0.19; 95% CI, −0.32 to 0.70, *I* ^2^ = NA). In the laparoscopic hysterectomy subgroup, the QL block was found superior in lowering pain score compared to the TAP block (SMD ‐5.03; 95% CI, −7.28 to −2.79, *I* ^2^ = 90%) and OSTAP (SMD ‐0.88; 95% CI, −1.37 to −0.38, *I* ^2^ = NA), but equivalent to placebo (SMD 0.27; 95% CI, −0.56 to 0.02, *I* ^2^ = NA) and ESP block (SMD 0.09; 95% CI, −0.45 to 0.62, *I* ^2^ = NA). (Supporting Data File—Supporting Figure 6a). A significant asymmetry in the funnel plot was observed (Egger’s regression test for funnel plot asymmetry: *Z* score = −5.650, *p* < 0.001) (Supporting Data File—Supporting Figure 6b). A sensitivity analysis based on the risk‐of‐bias assessment did not affect the overall findings (Supporting Data file—Supporting Table 2).

### 3.5. Safety Outcomes

Adverse events were reported in 14 studies (*n* = 1060) as postoperative nausea and vomiting (PONV). A trend of overall low incidence of PONV was found between the QL block and placebo (Supporting Data File—Supporting Figure 7a); however, no significant difference was found in other fascial plane blocks in either the laparoscopic or abdominal hysterectomy subgroup. The GRADE approach suggested a “low to moderate” quality of evidence (Table 4). No significant asymmetry in the funnel plot was observed (Egger’s regression test for funnel plot asymmetry: *Z* score = −0.082, *p* = 0.934) (Supporting Data File—Supporting Figure 7b). A sensitivity analysis based on the risk‐of‐bias assessment did not affect the overall findings (Supporting Data file—Supporting Table 2).

## 4. Discussion

In this meta‐analysis evaluating the QL block for postoperative analgesia following hysterectomy, we found that the QL block demonstrated either superior or comparable efficacy compared with other fascial plane blocks across several postoperative analgesic outcomes. Overall, the QL block prolonged the time to first rescue analgesic request, reduced postoperative opioid consumption within the first 24 h, and improved postoperative pain scores at several postoperative time intervals when compared to placebo and other fascial plane blocks. These findings suggest that the QL block may provide effective postoperative analgesia and may represent an important component of multimodal pain management in patients undergoing hysterectomy.

In the present meta‐analysis, the QL block demonstrated improved postoperative analgesia compared with placebo in several outcomes. In the abdominal hysterectomy subgroup, the QL block significantly prolonged the time to first rescue analgesia and reduced total analgesic consumption within the first 24 h. Similarly, postoperative pain scores were significantly lower during the early postoperative period. In laparoscopic hysterectomy, the QL block also reduced postoperative opioid consumption compared with placebo; however, the time to first rescue analgesia did not show a statistically significant difference, likely due to heterogeneity and the limited number of studies included in this subgroup. These results are consistent with recent systematic reviews evaluating the QL block in total laparoscopic hysterectomy which demonstrated that the QL block significantly reduced postoperative opioid consumption and improved postoperative pain scores at several early time points compared with placebo or no block [50].

The most consistent finding of our analysis was the superiority of the QL block compared with the TAP block. In both abdominal and laparoscopic hysterectomy subgroups, the QL block significantly prolonged the time to first rescue analgesia and reduced total 24‐h opioid consumption compared with the TAP block. The certainty of evidence for this outcome was rated as high for time to first rescue analgesia and moderate for total opioid consumption, according to the GRADE assessment.

Pain scores were also generally lower in the QL block group during early postoperative time intervals compared with the TAP block, although the certainty of evidence for pain score outcomes ranged from low to moderate due to heterogeneity across studies and potential publication bias. Earlier meta‐analyses have demonstrated that the QL block provides longer duration of analgesia and greater opioid‐sparing effects compared with the TAP block across a variety of abdominal and laparoscopic procedures [21, 51, 52]. More recent meta‐analysis including randomized trials in total abdominal hysterectomy also reported significantly lower postoperative pain scores at 12 and 24 h in patients receiving the QL block compared with the TAP block [53].

In the comparison between the QL block and the OSTAP block in laparoscopic hysterectomy, our analysis demonstrated superiority of the QL block in prolonging the time to first rescue analgesic request and reducing postoperative pain scores. The certainty of evidence for this comparison was rated as moderate for time to first rescue analgesia and opioid consumption based on the GRADE assessment. However, no statistically significant difference was observed in total 24‐h opioid consumption between the two techniques. The limited number of studies included in this comparison reduces the precision of the pooled estimate and highlights the need for further randomized trials directly comparing these regional techniques.

In abdominal hysterectomy, the QL block demonstrated comparable efficacy to the combined TAP block with TAP + IIN block (TAP + IIN). No statistically significant differences were observed between these techniques in terms of time to first rescue analgesia, postoperative pain scores, or total opioid consumption. The certainty of evidence for this comparison was rated as low, mainly due to the small number of studies available for this subgroup.

Our meta‐analysis also compared the QL block with the ESP block in laparoscopic hysterectomy. In this comparison, both techniques demonstrated comparable efficacy in terms of time to first rescue analgesia, postoperative opioid consumption, and pain scores. The certainty of evidence for this comparison was rated as moderate according to the GRADE assessment.

The higher analgesic efficacy of the QL block over TAP and OSTAP blocks may be explained by differences in their anatomical coverage and mechanisms of action. The QL block allows local anesthetic to spread along the TLF with potential extension into the paravertebral space, thereby affecting dorsal and ventral rami of spinal nerves as well as sympathetic fibers and providing both somatic and visceral analgesia [15–18, 54, 55]. This broader spread may result in wider dermatomal coverage and longer analgesic duration compared with superficial abdominal wall blocks, such as TAP and OSTAP blocks, which primarily block the anterior abdominal wall nerves (T6–L1) and therefore provide mainly somatic analgesia. Similarly, the comparable efficacy observed between the QL block and the ESP block may be related to their shared fascial plane mechanisms that permit paravertebral spread of local anesthetic [54, 55]. In addition, the comparable outcomes between the QL block and the combined TAP + IIN may be explained by the additional somatic nerve coverage provided by the IINs, which supply the lower abdominal wall and may enhance the analgesic coverage of the TAP block in lower abdominal surgeries.

The relative benefit of the QL block may also differ according to surgical approach. Abdominal hysterectomy involves a larger incision and greater somatic pain contribution, whereas laparoscopic hysterectomy is associated with smaller incisions and a greater visceral pain component related to pneumoperitoneum. Because the QL block may provide wider dermatomal coverage through TLF spread and potential paravertebral extension, it may offer broader somatic and visceral analgesia compared with more superficial abdominal wall blocks.

### 4.1. Safety Outcomes

With regard to safety outcomes, the incidence of PONV was comparable between the QL block and placebo as well as other fascial plane blocks. The certainty of evidence for PONV outcomes was rated as moderate according to the GRADE assessment.

While a statistically significant reduction in analgesic consumption ranging from approximately 2 mg–9 mg IV MME over 24 h was observed across various subgroups, this difference may not be clinically meaningful. The opioid‐sparing effect of any postoperative analgesic technique should translate into tangible clinical benefits as perceived by the patient. Currently, data are insufficient to define the minimum clinically important difference (MCID) for 24‐h opioid consumption [23, 52, 56]. Clinically meaningful outcomes would intuitively include reduced opioid use, fewer side effects, and improved functional recovery. No study has established the MCID for rescue opioid consumption in abdominal or laparoscopic hysterectomy. Previous meta‐analyses have also shown that the QL block is superior to no block or other blocks in reducing postoperative opioid use after renal, abdominal, and cesarean surgeries [17, 20, 51, 57]. Although our finding was in line with earlier studies, it should be reconfirmed by comparing the QL block with no block or placebo in hysterectomy with large sample size studies to draw any clinically relevant difference in total analgesic consumption.

### 4.2. Clinical Implications

From a clinical perspective, the choice of regional analgesic technique should consider not only analgesic efficacy but also technical feasibility, safety profile, and operator experience. Although the QL block may provide broader analgesic coverage compared with TAP or OSTAP blocks, it requires deeper needle placement and greater technical expertise. Alternative strategies, such as continuous wound infusion techniques, have also demonstrated comparable analgesic efficacy following abdominal hysterectomy and may offer practical advantages in selected clinical settings [58]. Therefore, individualized analgesic strategies tailored to surgical approach, patient characteristics, and institutional expertise are essential when incorporating regional techniques into multimodal analgesia protocols.

Furthermore, the QL block may have an important role within enhanced recovery after surgery (ERAS) protocols for hysterectomy patients. Regional analgesic techniques that reduce opioid consumption and improve postoperative pain control may facilitate earlier mobilization, faster recovery, and improved patient satisfaction. The American College of Obstetricians and Gynecologists has emphasized the importance of multimodal analgesia within ERAS pathways for gynecological surgery [59]. However, evidence regarding the impact of the QL block on broader recovery outcomes, such as hospital length of stay, quality of recovery, and long‐term patient‐reported outcomes, remains limited and warrants further investigation.

Given these findings, the results of this meta‐analysis should be interpreted in the context of the available evidence and the limitations of the included studies.

### 4.3. Limitations

Several limitations should be considered when interpreting the findings of this meta‐analysis. Although 15 RCTs involving 1130 patients were included, the effective sample size within each subgroup remained relatively small because studies were divided according to surgical approach (eight laparoscopic and seven abdominal hysterectomy) and comparator interventions. Additionally, six studies were judged to have “some concerns” according to the ROB‐II tool.

Most included trials were conducted in Asian populations, which may limit the generalizability of the findings to other ethnic groups. Considerable clinical heterogeneity was also observed due to variations in patient demographics, surgical techniques, anesthesia methods, timing of block administration, and comparator interventions (placebo, TAP, combined TAP with IIN block, OSTAP, and ESP block).

Variations in QL block techniques may have influenced analgesic outcomes. Previous studies suggest that the anterior (transmuscular) QL block approach may provide superior postoperative analgesia and lower opioid consumption compared with posterior and lateral approaches because of wider spread of injectate [54, 55]. In the included trials, the lateral approach was used in one study [31], the posterior approach in five studies [36–38, 40, 43], the anterior (transmuscular) approach in seven studies [30, 32–35, 39, 41], a combined posterior and anterior approach in one study [42], and one study did not specify the block technique. However, subgroup analysis according to block approach was not feasible due to insufficient data.

Further heterogeneity may have resulted from variations in the type, concentration, and volume of local anesthetics used across studies; therefore, random‐effects models were applied. Two studies (Midathala et al. and Kothapalli et al.) also used dexmedetomidine as an adjuvant in both QL and TAP block groups during laparoscopic hysterectomy, and sensitivity analysis excluding these studies was not feasible.

Publication bias was detected for outcomes including analgesic consumption and pain scores. In addition, postoperative analgesic protocols varied across studies, with multimodal analgesics (paracetamol, fentanyl, morphine, diclofenac, pethidine, flurbiprofen, and ibuprofen) administered either at fixed intervals or through intravenous patient‐controlled analgesia, which may have influenced pain assessment and opioid consumption.

Finally, pain outcomes were reported using different scales and time points across studies. In some cases, median and interquartile range values were converted to means and standard deviations, and pain scores were analyzed across broader time intervals rather than specific time points. Data from three‐arm trials were used only for subgroup analyses to avoid overestimation of treatment effects.

Despite these limitations, the results remained broadly consistent across subgroup and sensitivity analyses, supporting the overall robustness of the findings.

## 5. Conclusion

High‐ to low‐quality evidence suggests that the QL block offers effective postoperative analgesia in patients undergoing abdominal and laparoscopic hysterectomy. The QL block provides superior analgesia compared with the TAP and OSTAP blocks. It shows comparable efficacy with the ESP block in laparoscopic hysterectomy. Our findings should be interpreted cautiously due to the possibility of publication bias in analgesic consumption and pain score outcomes. Further large‐scale trials are warranted to confirm our findings and establish the optimal block technique, dosage, and timing of QL block administration and its impact on the quality of recovery in hysterectomy patients.

## Author Contributions

Priyanka Dwivedi: conceptualization, data curation, formal analysis, investigation, and writing–original draft. Tejas K. Patel: conceptualization, methodology, supervision, writing–review and editing, and data verification. Vijeta Bajpai: data curation, investigation, and writing–original draft. Chanchal Goyal: methodology and writing–review and editing. Ankita Kabi: data curation and cross‐verification of data extraction. Santosh Kumar Sharma: writing–review and editing. Hari Shanker Joshi: methodology and writing–review and editing. Pratibha Singh: writing–review and editing.

## Funding

This research received no specific grant from any funding agency in the public, commercial, or not‐for‐profit sectors.

## Ethics Statement

Ethics approval was not required for this study, as it is a systematic review and meta‐analysis of previously published data and does not involve direct patient participation.

## Conflicts of Interest

The authors declare no conflicts of interest.

## Supporting Information

Additional supporting information can be found online in the Supporting Information section.

## Supporting information


**Supporting Information** Supporting Data file: Supporting Figure 1: Risk of bias assessment among included studies. Supporting Figure 2a: Funnel plot—time to first rescue analgesia. Supporting Figure 2b: Funnel plot—total analgesic consumption in 24 h. Supporting Figure 3a: Meta‐analytic summary of pain score at 0–2 h after interventions. Supporting Figure 3b: Funnel plot—pain score at 0–2 h after interventions. Supporting Figure 4a: Meta‐analytic summary of pain score at 2–6 h after interventions. Supporting Figure 4b: Funnel plot—pain score at 2–6 h after interventions. Supporting Figure 5a: Meta‐analytic summary of pain score at 6–12 h after interventions. Supporting Figure 5b: Funnel plot—pain score at 6–12 h after interventions. Supporting Figure 6a: Meta‐analytic summary of pain score at 12–24 h after interventions. Supporting Figure 6b: Funnel plot—pain score at 12–24 h after interventions. Supporting Figure 7a: Meta‐analytic summary of the postoperative nausea and vomiting through a random effect model. Supporting Figure 7b: Funnel plot—postoperative nausea and vomiting. Supporting Table 1: Opioid conversion table for IV morphine milligram equivalent (MME). Supporting Table 2: Meta‐analytic summary of all outcomes in all studies and low risk of bias studies. Supporting Table 3: Univariate regression for the primary outcome—time to first rescue analgesia. Supporting Table 4: 24‐h Total analgesic consumption in morphine milligram equivalent (MME).

## Data Availability

The data that support the findings of this study are available in the Supporting Information of this article.
