# Supplementary material for: Comparative Efficacy of Ultrasound‐Guided Quadratus Lumborum Block Versus Alternative Fascial Plane Blocks for Postoperative Analgesia Following Hysterectomy: A Systematic Review and Meta‐Analysis
Source: Anesthesiol Res Pract. 2026 May 17;2026:5760922. doi: 10.1155/anrp/5760922 (PMC13181214; doi:10.1155/anrp/5760922)
Supplement: Supplementary file 1 — Supporting Information Supporting Data file: Supporting Figure 1: Risk of bias assessment among included studies. Supporting Figure 2a: Funnel plot—time to first rescue analgesia. Supporting Figure 2b: Funnel plot—total analgesic consumption in 24 h. Supporting Figure 3a: Meta‐analytic summary of pain score at 0–2 h after interventions. Supporting Figure 3b: Funnel plot—pain score at 0–2 h after interventions. Supporting Figure 4a: Meta‐analytic summary of pain score at 2–6 h after interventions. Supporting Figure 4b: Funnel plot—pain score at 2–6 h after interventions. Supporting Figure 5a: Meta‐analytic summary of pain score at 6–12 h after interventions. Supporting Figure 5b: Funnel plot—pain score at 6–12 h after interventions. Supporting Figure 6a: Meta‐analytic summary of pain score at 12–24 h after interventions. Supporting Figure 6b: Funnel plot—pain score at 12–24 h after interventions. Supporting Figure 7a: Meta‐analytic summary of the postoperative nausea and vomiting through a random effect model. Supporting Figure 7b: Funnel plot—postoperative nausea and vomiting. Supporting Table 1: Opioid conversion table for IV morphine milligram equivalent (MME). Supporting Table 2: Meta‐analytic summary of all outcomes in all studies and low risk of bias studies. Supporting Table 3: Univariate regression for the primary outcome—time to first rescue analgesia. Supporting Table 4: 24‐h Total analgesic consumption in morphine milligram equivalent (MME). [file ANRP-2026-5760922-s001.docx]

Supplementary Figure 1: Risk of bias assessment among included studies


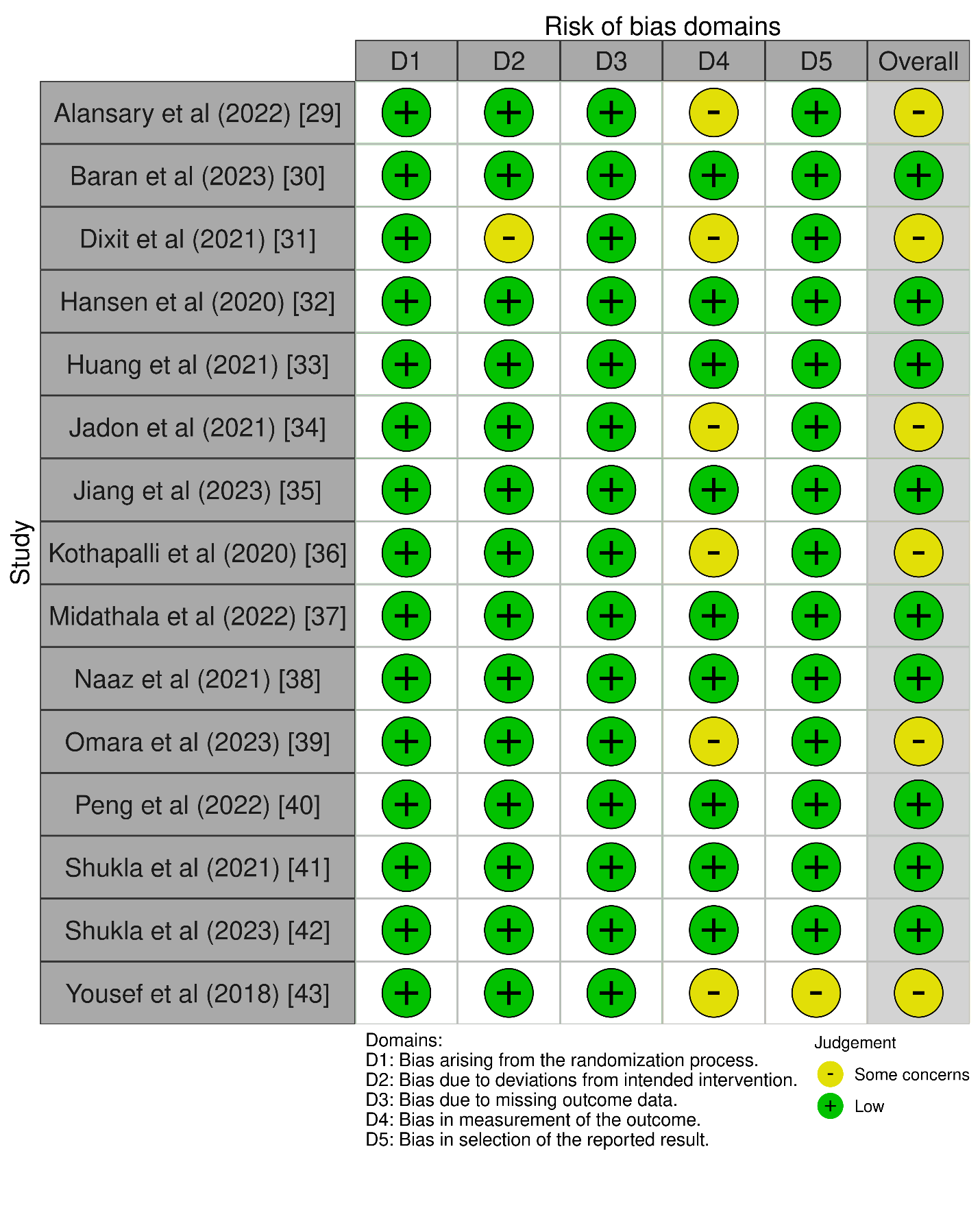


Supplementary Figure 2a: Funnel plot – Time to first rescue analgesia


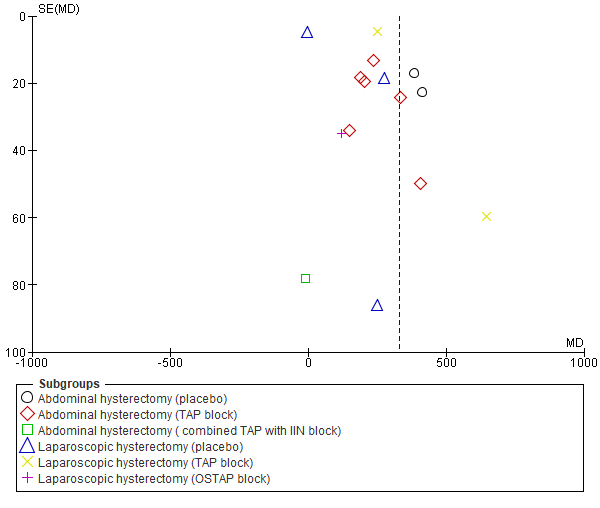


Abbreviations: QL block – Quadratus Lumborum Block; TAP block – Transversus Abdominis Plane Block; IIN block – Ilioinguinal Nerve Block; OSTAP block – Oblique Subcostal Transversus Abdominis Plane Block; ESP block – Erector Spinae Plane Block.

Supplementary Figure 2b: Funnel plot – Total analgesic consumption in 24 hr


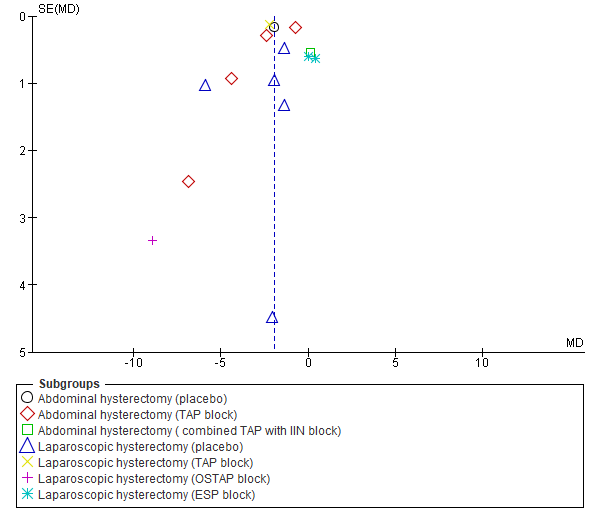


Abbreviations: QL block – Quadratus Lumborum Block; TAP block – Transversus Abdominis Plane Block; IIN block – Ilioinguinal Nerve Block; OSTAP block – Oblique Subcostal Transversus Abdominis Plane Block; ESP block – Erector Spinae Plane Block.

Supplementary Figure 3a: Meta-analytic summary of Pain score at 0-2 hr after interventions


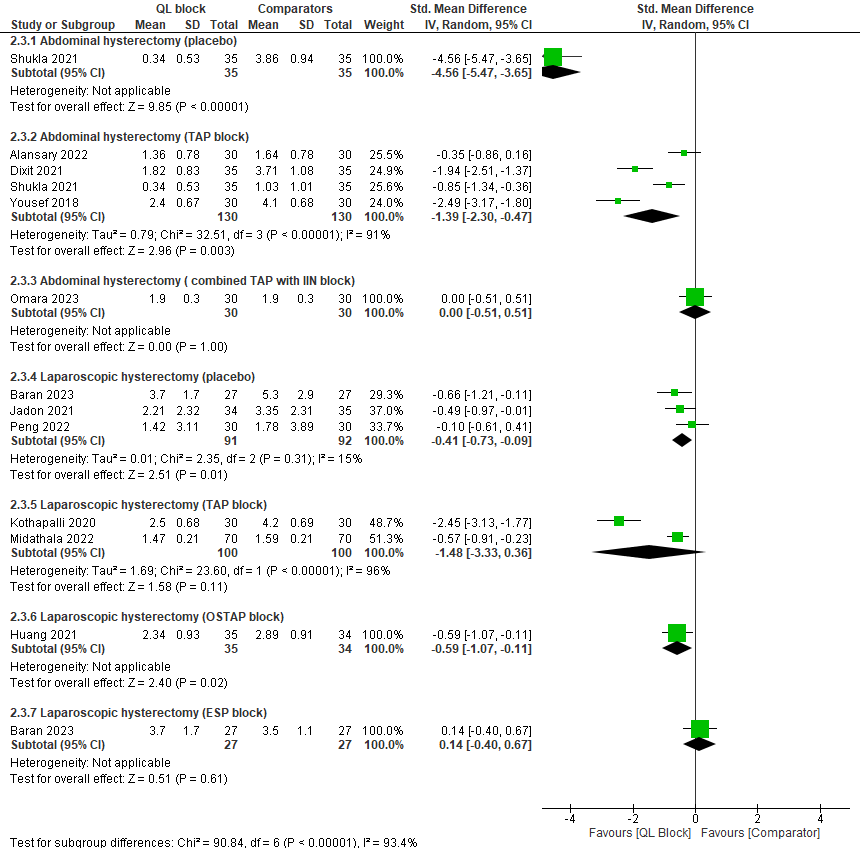


Abbreviations: QL block – Quadratus Lumborum Block; TAP block – Transversus Abdominis Plane Block; IIN block – Ilioinguinal Nerve Block; OSTAP block – Oblique Subcostal Transversus Abdominis Plane Block; ESP block – Erector Spinae Plane Block; SD – Standard Deviation; CI – Confidence Interval; IV – Inverse Variance.

Supplementary Figure 3b: Funnel plot – Pain score at 0-2 hr after interventions


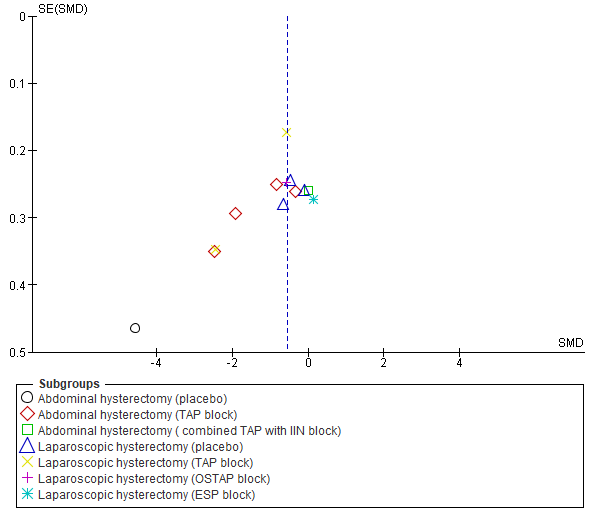


Abbreviations: QL block – Quadratus Lumborum Block; TAP block – Transversus Abdominis Plane Block; IIN block – Ilioinguinal Nerve Block; OSTAP block – Oblique Subcostal Transversus Abdominis Plane Block; ESP block – Erector Spinae Plane Block.

Supplementary Figure 4a: Meta-analytic summary of Pain score at 2-6 hr after interventions


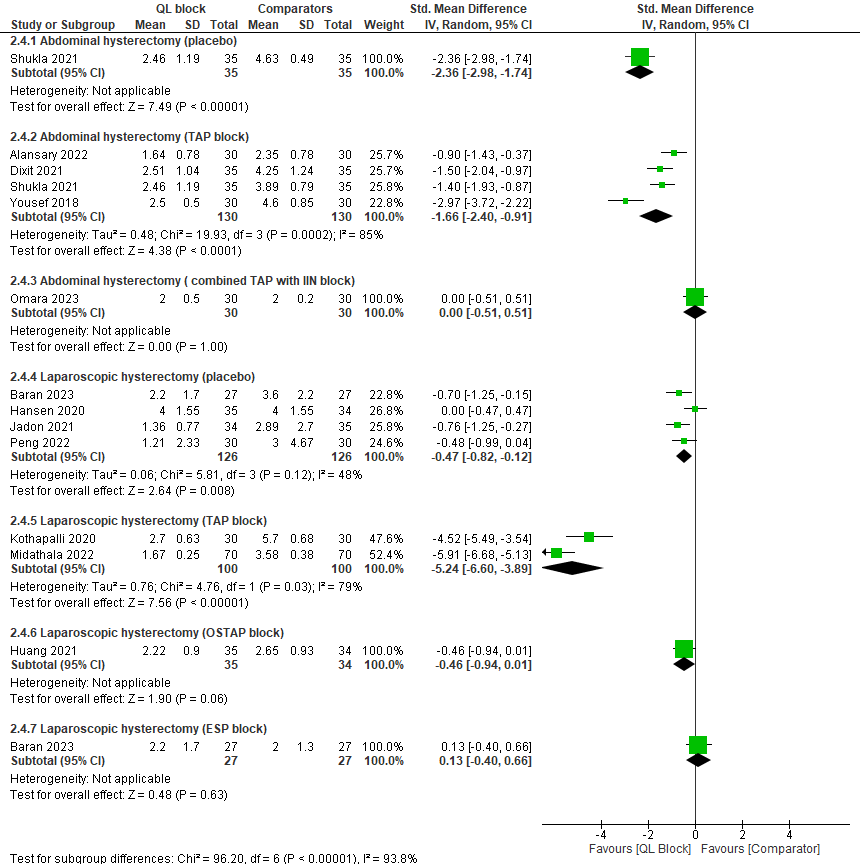


Abbreviations: QL block – Quadratus Lumborum Block; TAP block – Transversus Abdominis Plane Block; IIN block – Ilioinguinal Nerve Block; OSTAP block – Oblique Subcostal Transversus Abdominis Plane Block; ESP block – Erector Spinae Plane Block; SD – Standard Deviation; CI – Confidence Interval; IV – Inverse Variance.

Supplementary Figure 4b: Funnel plot – Pain score at 2-6 hr after interventions


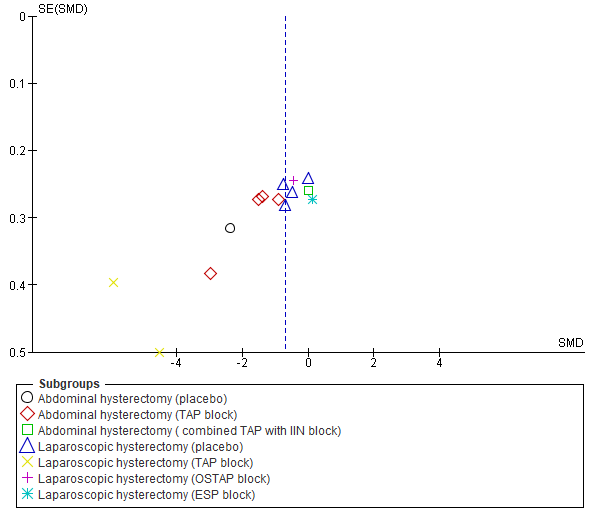


Abbreviations: QL block – Quadratus Lumborum Block; TAP block – Transversus Abdominis Plane Block; IIN block – Ilioinguinal Nerve Block; OSTAP block – Oblique Subcostal Transversus Abdominis Plane Block; ESP block – Erector Spinae Plane Block.

Supplementary Figure 5a: Meta-analytic summary of Pain score at 6-12 hr after interventions


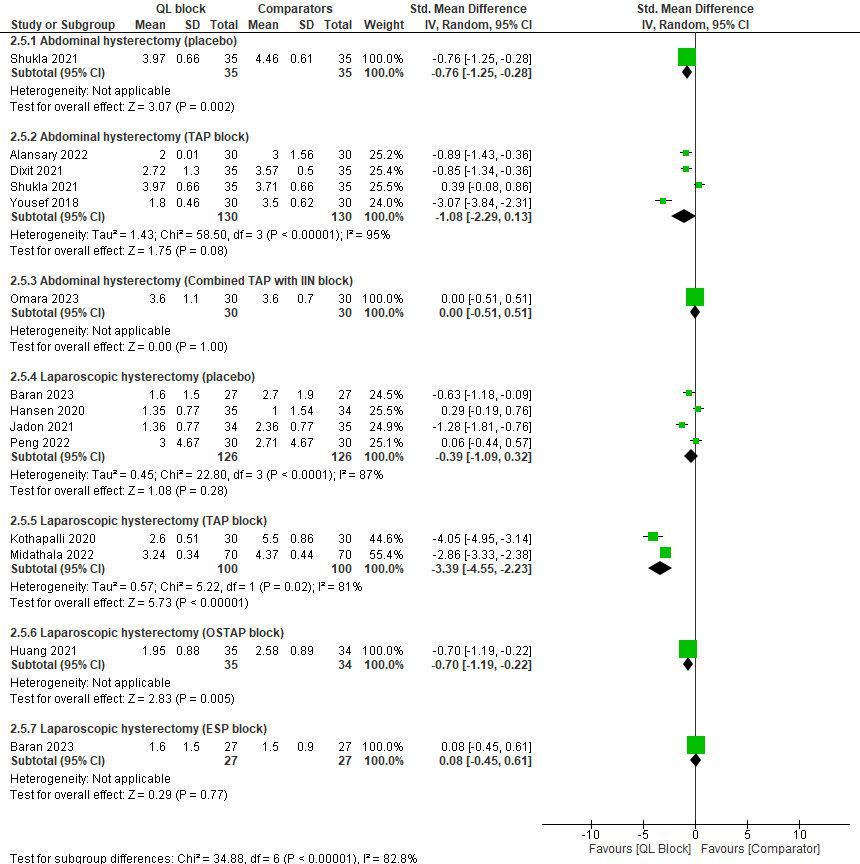


Abbreviations: QL block – Quadratus Lumborum Block; TAP block – Transversus Abdominis Plane Block; IIN block – Ilioinguinal Nerve Block; OSTAP block – Oblique Subcostal Transversus Abdominis Plane Block; ESP block – Erector Spinae Plane Block; SD – Standard Deviation; CI – Confidence Interval; IV – Inverse Variance.

Supplementary Figure 5b: Funnel plot – Pain score at 6-12 hr after interventions


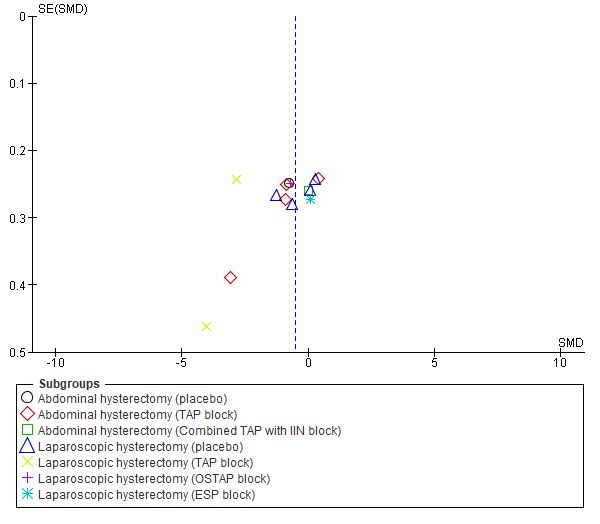


Abbreviations: QL block – Quadratus Lumborum Block; TAP block – Transversus Abdominis Plane Block; IIN block – Ilioinguinal Nerve Block; OSTAP block – Oblique Subcostal Transversus Abdominis Plane Block; ESP block – Erector Spinae Plane Block.

Supplementary Figure 6a: Meta-analytic summary of Pain score at 12-24 hr after interventions


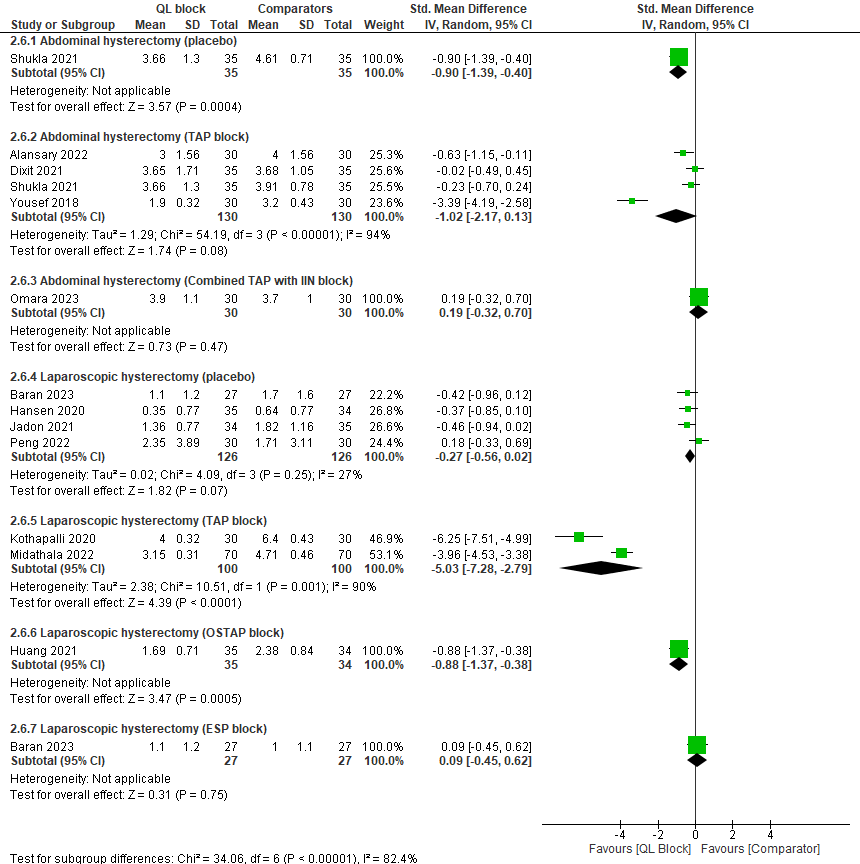


Abbreviations: QL block – Quadratus Lumborum Block; TAP block – Transversus Abdominis Plane Block; IIN block – Ilioinguinal Nerve Block; OSTAP block – Oblique Subcostal Transversus Abdominis Plane Block; ESP block – Erector Spinae Plane Block; SD – Standard Deviation; CI – Confidence Interval; IV – Inverse Variance.

Supplementary Figure 6b: Funnel plot – Pain score at 12-24 hr after interventions


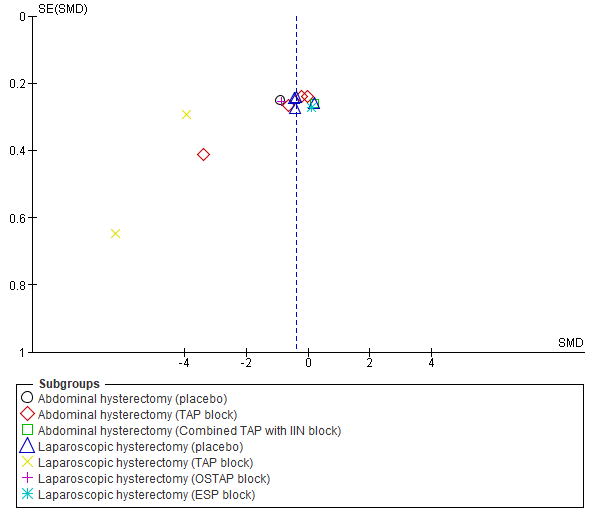


Abbreviations: QL block – Quadratus Lumborum Block; TAP block – Transversus Abdominis Plane Block; IIN block – Ilioinguinal Nerve Block; OSTAP block – Oblique Subcostal Transversus Abdominis Plane Block; ESP block – Erector Spinae Plane Block.

Supplementary Figure 7a: Meta-analytic summary of the postoperative nausea and vomiting through a random effect model


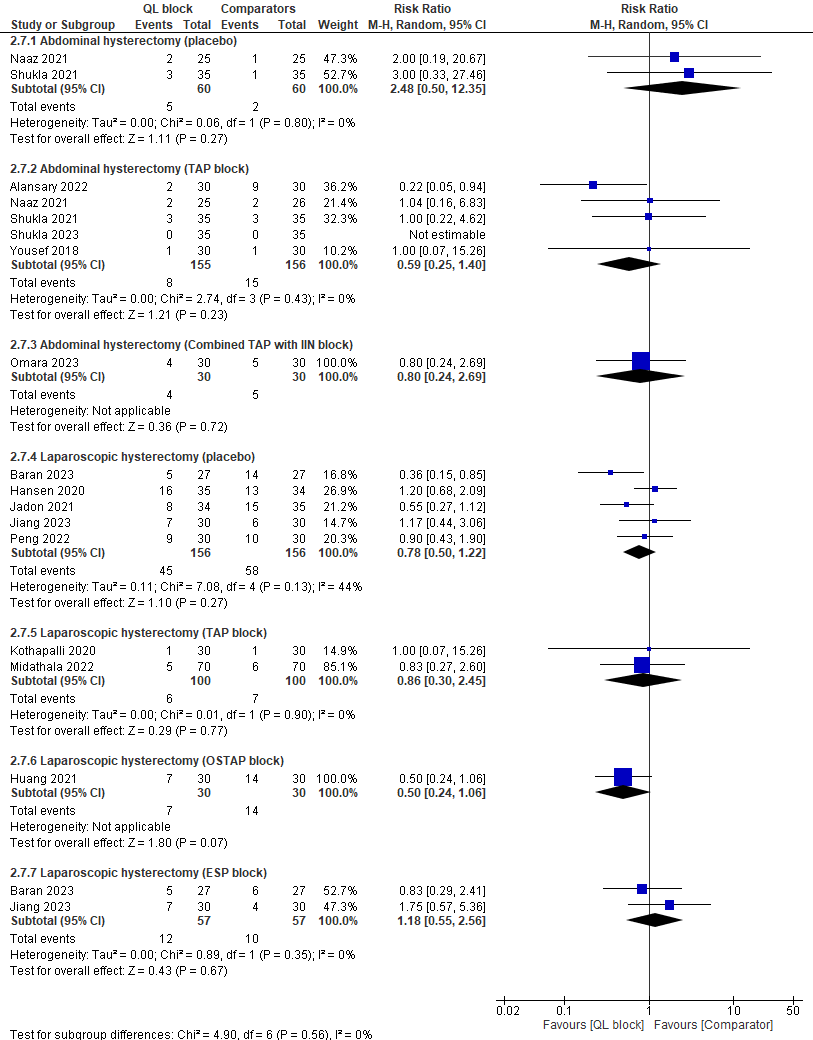


Abbreviations: QL block – Quadratus Lumborum Block; TAP block – Transversus Abdominis Plane Block; IIN block – Ilioinguinal Nerve Block; OSTAP block – Oblique Subcostal Transversus Abdominis Plane Block; ESP block – Erector Spinae Plane Block; RR – Risk ratio; CI – Confidence Interval; M-H – Mantle Hanzle.

Supplementary Figure 7b: Funnel plot – Postoperative nausea and vomiting


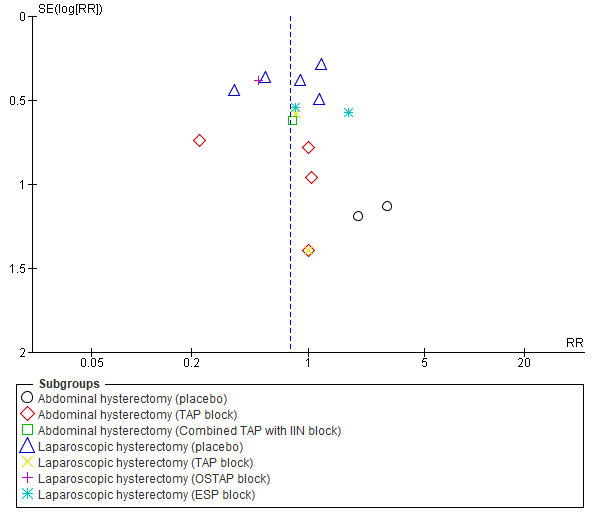


Abbreviations: QL block – Quadratus Lumborum Block; TAP block – Transversus Abdominis Plane Block; IIN block – Ilioinguinal Nerve Block; OSTAP block – Oblique Subcostal Transversus Abdominis Plane Block; ESP block – Erector Spinae Plane Block.

**Supplementary Table 1: Opioid conversion table for IV morphine milligram equivalent (MME)**

| **Opioid** | **Route** | **Oral morphine equivalents** | **Conversion factor for oral MME** | **Conversion factor for IV MME** |
| --- | --- | --- | --- | --- |
| Morphine^1^ | 1 mg PO | 1 mg | 1 mg | 1/3 mg |
| Morphine1 | 1 mg IV | 3 mg | 3 mg | 3/3= 1 mg |
| Pethidine^2^ | 1 mg IV | 0.3 mg | 0.3 mg | 0.3/3= 0.1 mg |
| Tramadol^2^ | 1mg IV | 0.3 mg | 0.3 mg | 0.3/3=0.1 mg |
| Fentanyl^3^ | 1 mcg IV | 300 mcg or 0.3 mg | 0.3 mg | 0.3/3= 0.1 mg |
| Sufentanil | 1 mcg IV | 3000 mcg or 3 mg | 3 mg | 3/3= 1 mg |

1. Nielsen S, Degenhardt L, Hoban B, Gisev N. A synthesis of oral morphine equivalents (OME) for opioid utilisation studies. *Pharmacoepidemiol Drug Saf*. 2016;25(6):733-737. doi:10.1002/pds.394
2. Unlugenc H, Vardar MA, Tetiker S. A comparative study of the analgesic effect of patient-controlled morphine, pethidine, and tramadol for postoperative pain management after abdominal hysterectomy. Anesth Analg. 2008 Jan;106(1):309-12, table of contents.
3. Tan S, Lee E, Lee S, Sakaria SS, Roh JS. Morphine Equianalgesic Dose Chart in the Emergency Department. J Educ Teach Emerg Med. 2022 Jul 15;7(3):L1-L20. doi: 10.21980/J8RD29. PMID: 37465775; PMCID: PMC10332699.
4. World Health Organization. WHO guidelines for the pharmacological and radiotherapeutic management of cancer pain in adults and adolescents. World Health Organization; 2018.

**Supplementary Table 2: Meta-analytic summary of all outcomes in all studies and low risk of bias studies**

|  | **All studies** | | | | **Low risk of bias studies** | | | |
| --- | --- | --- | --- | --- | --- | --- | --- | --- |
| **Outcomes** | **Studies** | **Participants** | **Effect estimate**  **MD (95% CI)/ SMD (95% CI) */ RR (95% CI) **** | **I²** | **Studies** | **Participants** | **Effect estimate**  **MD (95% CI)/ SMD (95% CI) */ RR (95% CI) **** | **I²** |
| **Time for 1^st^ rescue analgesia** | **13** |  | **Subtotals only** |  | **7** | **Subtotals only** |  |  |
| Abdominal hysterectomy (Placebo) | 2 | 120 | 393.99 [367.29, 420.69] | 0% | 2 | 120 | 393.99 [367.29, 420.69] | 0% |
| Abdominal hysterectomy (TAP block) | 6 | 381 | 244.77 [191.98, 297.56] | 89% | 3 | 191 | 224.03 [117.70, 330.35] | 93% |
| Abdominal hysterectomy (Combined TAP with IIN block) | 1 | 60 | -12.00 [-164.91, 140.91] | NA | 0 | 0 | Not estimable | NA |
| Laparoscopic hysterectomy (Placebo) | 3 | 192 | 169.26 [-59.48, 398.00] | 99% | 2 | 123 | 108.34 [-139.37, 356.06] | 89% |
| Laparoscopic hysterectomy (TAP block) | 2 | 200 | 443.15 [56.37, 829.93] | 98% | 1 | 140 | 250.22 [241.47, 258.97] | NA |
| Laparoscopic hysterectomy (OSTAP block) | 1 | 60 | 120.00 [51.46, 188.54] | NA | 1 | 60 | 120.00 [51.46, 188.54] | NA |
| Laparoscopic hysterectomy (ESP block) | 1 | 54 | -20.00 [-194.79, 154.79] | NA | 1 | 54 | -20.00 [-194.79, 154.79] | NA |
| **Analgesic consumption in 24 hours** | **12** |  | **Subtotals only** |  | **7** | **Subtotals only** |  |  |
| Abdominal hysterectomy (Placebo) | 1 | 50 | -1.94 [-2.26, -1.62] | NA | 1 | 50 | -1.94 [-2.26, -1.62] | NA |
| Abdominal hysterectomy (TAP block) | 4 | 241 | -2.64 [-4.19, -1.09] | 93% | 1 | 51 | -0.74 [-1.06, -0.42] | NA |
| Abdominal hysterectomy (Combined TAP with IIN block) | 1 | 60 | 0.10 [-0.97, 1.17] | NA | 0 | 0 | Not estimable | NA |
| Laparoscopic hysterectomy (Placebo) | 5 | 312 | -3.82 [-5.77, -1.88] | 47% | 4 | 243 | -2.82 [-4.50, -1.14] | 0% |
| Laparoscopic hysterectomy (TAP block) | 1 | 140 | -6.65 [-7.39, -5.91] | NA | 1 | 140 | -6.65 [-7.39, -5.91] | NA |
| Laparoscopic hysterectomy (OSTAP block) | 1 | 60 | -8.90 [-15.43, -2.37] | NA | 1 | 60 | -8.90 [-15.43, -2.37] | NA |
| Laparoscopic hysterectomy (ESP block) | 2 | 114 | 0.34 [-1.65, 2.33] | 0% | 2 | 114 | 0.34 [-1.65, 2.33] | 0% |
| **Pain score (0-2 hrs)** | **11** |  | **Subtotals only** |  | **5** | **Subtotals only** |  |  |
| Abdominal hysterectomy (Placebo) | 1 | 70 | -4.56 [-5.47, -3.65] ***** | NA | 1 | 70 | -4.56 [-5.47, -3.65] ***** | NA |
| Abdominal hysterectomy (TAP block) | 4 | 260 | -1.39 [-2.30, -0.47] ***** | 91% | 1 | 70 | -0.85 [-1.34, -0.36] ***** | NA |
| Abdominal hysterectomy (Combined TAP with IIN block) | 1 | 60 | 0.00 [-0.51, 0.51] ***** | NA | 0 | 0 | Not estimable | NA |
| Laparoscopic hysterectomy (Placebo) | 3 | 183 | -0.41 [-0.73, -0.09] ***** | 15% | 2 | 114 | -0.37 [-0.92, 0.18] ***** | 54% |
| Laparoscopic hysterectomy (TAP block) | 2 | 200 | -1.48 [-3.33, 0.36] ***** | 96% | 1 | 140 | -0.57 [-0.91, -0.23] ***** | NA |
| Laparoscopic hysterectomy (OSTAP block) | 1 | 69 | -0.59 [-1.07, -0.11] ***** | NA | 1 | 69 | -0.59 [-1.07, -0.11] ***** | NA |
| Laparoscopic hysterectomy (ESP block) | 1 | 54 | 0.14 [-0.40, 0.67] ***** | NA | 1 | 54 | 0.14 [-0.40, 0.67] ***** | NA |
| **Pain score (2-6 hrs)** | **12** |  | **Subtotals only** |  | **6** | **Subtotals only** |  |  |
| Abdominal hysterectomy (Placebo) | 1 | 70 | -2.36 [-2.98, -1.74] * | NA | 1 | 70 | -2.36 [-2.98, -1.74] * | NA |
| Abdominal hysterectomy (TAP block) | 4 | 260 | -1.66 [-2.40, -0.91] ***** | 85% | 1 | 70 | -1.40 [-1.93, -0.87] ***** | NA |
| Abdominal hysterectomy (Combined TAP with IIN block) | 1 | 60 | 0.00 [-0.51, 0.51] ***** | NA | 0 | 0 | Not estimable | NA |
| Laparoscopic hysterectomy (Placebo) | 4 | 252 | -0.47 [-0.82, -0.12] ***** | 48% | 3 | 183 | -0.37 [-0.79, 0.04] ***** | 49% |
| Laparoscopic hysterectomy (TAP block) | 2 | 200 | -5.24 [-6.60, -3.89] ***** | 79% | 1 | 140 | -5.91 [-6.68, -5.13] ***** | NA |
| Laparoscopic hysterectomy (OSTAP block) | 1 | 69 | -0.46 [-0.94, 0.01] ***** | NA | 1 | 69 | -0.46 [-0.94, 0.01] ***** | NA |
| Laparoscopic hysterectomy (ESP block) | 1 | 54 | 0.13 [-0.40, 0.66] * | NA | 1 | 54 | 0.13 [-0.40, 0.66] * | NA |
| **Pain score (6-12 hrs)** | **12** |  | **Subtotals only** |  | **3** | **Subtotals only** |  |  |
| Abdominal hysterectomy (Placebo) | 1 | 70 | -0.76 [-1.25, -0.28] * | NA | 1 | 70 | -0.76 [-1.25, -0.28] * | NA |
| Abdominal hysterectomy (TAP block) | 4 | 260 | -1.08 [-2.29, 0.13] ***** | 95% | 1 | 70 | 0.39 [-0.08, 0.86] ***** | NA |
| Abdominal hysterectomy (Combined TAP with IIN block) | 1 | 60 | 0.00 [-0.51, 0.51] ***** | NA | 0 | 0 | Not estimable | NA |
| Laparoscopic hysterectomy (Placebo) | 4 | 252 | -0.39 [-1.09, 0.32] * | 87% | 3 | 183 | -0.08 [-0.61, 0.45] ***** | 69% |
| Laparoscopic hysterectomy (TAP block) | 2 | 200 | -3.39 [-4.55, -2.23] ***** | 81% | 1 | 140 | -2.86 [-3.33, -2.38] ***** | NA |
| Laparoscopic hysterectomy (OSTAP block) | 1 | 69 | -0.70 [-1.19, -0.22] ***** | NA | 1 | 69 | -0.70 [-1.19, -0.22] ***** | NA |
| Laparoscopic hysterectomy (ESP block) | 1 | 54 | 0.08 [-0.45, 0.61] * | NA | 1 | 54 | 0.08 [-0.45, 0.61] 1* | NA |
| **Pain score (12-24 hrs)** | **12** |  | **Subtotals only** |  | **3** | **Subtotals only** |  |  |
| Abdominal hysterectomy (Placebo) | 1 | 70 | -0.90 [-1.39, -0.40] * | NA | 1 | 70 | -0.90 [-1.39, -0.40] * | NA |
| Abdominal hysterectomy (TAP block) | 4 | 260 | -1.02 [-2.17, 0.13] ***** | 94% | 1 | 70 | -0.23 [-0.70, 0.24] ***** | NA |
| Abdominal hysterectomy (Combined TAP with IIN block) | 1 | 60 | 0.19 [-0.32, 0.70] ***** | NA | 0 | 0 | Not estimable | NA |
| Laparoscopic hysterectomy (Placebo) | 4 | 252 | -0.27 [-0.56, 0.02] * | 27% | 3 | 183 | -0.20 [-0.58, 0.17] ***** | 39% |
| Laparoscopic hysterectomy (TAP block) | 2 | 200 | -5.03 [-7.28, -2.79] ***** | 90% | 1 | 140 | -3.96 [-4.53, -3.38] ***** | NA |
| Laparoscopic hysterectomy (OSTAP block) | 1 | 69 | -0.88 [-1.37, -0.38] ***** | NA | 1 | 69 | -0.88 [-1.37, -0.38] ***** | NA |
| Laparoscopic hysterectomy (ESP block) | 1 | 54 | 0.09 [-0.45, 0.62] * | NA | 1 | 54 | 0.09 [-0.45, 0.62] ***** | NA |
| **Post operative nausea and vomiting** | **14** |  | **Subtotals only** |  | **8** |  | **Subtotals only** |  |
| Abdominal hysterectomy (Placebo) | 2 | 120 | 2.48 [0.50, 12.35] ** | 0% | 2 | 120 | 2.48 [0.50, 12.35] ** | 0% |
| Abdominal hysterectomy (TAP block) | 5 | 311 | 0.59 [0.25, 1.40] ****** | 0% | 3 | 191 | 1.02 [0.31, 3.33] ****** | 0% |
| Abdominal hysterectomy (Combined TAP with IIN block) | 1 | 60 | 0.80 [0.24, 2.69] ****** | NA | 0 | 0 | Not estimable | NA |
| Laparoscopic hysterectomy (Placebo) | 5 | 312 | 0.78 [0.50, 1.22] ** | 44% | 3 | 243 | 0.85 [0.50, 1.44] ****** | 47% |
| Laparoscopic hysterectomy (TAP block) | 2 | 200 | 0.86 [0.30, 2.45] ****** | 0% | 1 | 140 | 0.83 [0.27, 2.60] ****** | NA |
| Laparoscopic hysterectomy (OSTAP block) | 1 | 60 | 0.50 [0.24, 1.06] ****** | NA | 1 | 60 | 0.50 [0.24, 1.06] ****** | NA |
| Laparoscopic hysterectomy (ESP block) | 2 | 114 | 1.18 [0.55, 2.56] ** | 0% | 1 | 54 | 0.83 [0.29, 2.41] ** | NA |

MD - mean difference; SMD - standardized mean difference; RR – risk ratio; CI - confidence interval.

TAP - Transabdominal Plane; IIN - ilioinguinal and iliohypogastric nerves; OSTAP - Oblique subcostal transversus abdominis plane block.

**Supplementary Table 3:** Univariate regression for the primary outcome - time to first rescue analgesia

| Variable | Estimate | 95% CI | SE | P- Value |
| --- | --- | --- | --- | --- |
| Types of comparators |  |  |  |  |
| Intercept | 120.00 | (-236.12, 476.12) | 161.80 | 0.474 |
| Placebo | 143.18 | (-247.21, 533.57) | 177.37 | 0.437 |
| TAP block | 175.54 | (-202.03, 553.11) | 171.55 | 0.328 |
| TAP with IIN block | 132.00 | (-658.51, 394.51) | 239.22 | 0.592 |
| Types of surgery |  |  |  |  |
| Intercept | 259.64 | (141.04, 378.24) | 54.90 | < 0.001 |
| Laparoscopic hysterectomy | -10.52 | (-199.35, 178.30) | 87.40 | 0.906 |
| Types of anaesthesia |  |  |  |  |
| Intercept | 269.48 | (155.15, 383.81) | 52.92 | < 0.001 |
| Spinal anaesthesia | -41.79 | (-239.43, 155.86) | 91.49 | 0.655 |
| Types of drugs for block |  |  |  |  |
| Intercept | 262.57 | (151.40, 373.73) | 50.51 | < 0.001 |
| Ropivacaine | 132.58 | (-349.91, 84.74) | 98.74 | 0.206 |
| Bupivacaine and Dexmedetomidine | 169.85 | (-89.76, 429.45) | 117.95 | 0.178 |

**Supplementary Table 4:** **24 hours Total analgesic consumption in morphine milligram equivalent (MME)**

| **Study Name** | **Group** | **Opioid** | **Mean** | **SD** | **Morphine** | **mean (ME)** | **SD (ME)** |
| --- | --- | --- | --- | --- | --- | --- | --- |
| Alansary et al (2022) (29) | QL | Pethidine | 68.33 | 66.28 | 0.1 | 6.83 | 6.63 |
|  | TAP | Pethidine | 120 | 76.11 | 0.1 | 12.00 | 7.61 |
| Baran et al (2023) (30) | QL | Tramadol | 190.4 | 61.7 | 0.1 | 19.04 | 6.17 |
|  | ESPB | Tramadol | 178.7 | 75.7 | 0.1 | 17.87 | 7.57 |
|  | Placebo | Tramadol | 230.9 | 44.3 | 0.1 | 23.09 | 4.43 |
| Dixit et al (2021) (31) | QL | Morphine IV | 3.23 | 0.72 | 1 | 3.23 | 0.72 |
|  | TAP | Morphine IV | 5.63 | 1.52 | 1 | 5.63 | 1.52 |
| Hansen et al (2020) (32) | Ql | Morphine oral | 62.7 | 52.9 | 1/3 | 20.90 | 17.63 |
|  | Placebo | Morphine oral | 68.9 | 58.3 | 1/3 | 22.97 | 19.43 |
| Huang et al (2021) (33) | Ql | Morphine IV | 17.2 | 12.5 | 1 | 17.20 | 12.50 |
|  | TAP | Morphine IV | 26.1 | 13.3 | 1 | 26.10 | 13.30 |
| Jadon et al (2021) (22) | QL | Fentanyl | 0.1673 | 0.044 | 0.1 | 16.73 | 4.40 |
|  | Placebo | Fentanyl | 0.2265 | 0.0419 | 0.1 | 22.65 | 4.19 |
| Jiang et al (2023) (34) | QL | Sufentanyl PCA | 2.139 | 4.67 | 1 | 2.139 | 4.67 |
|  | ESPB | Sufentanyl PCA | 2.139 | 4.67 | 1 | 2.139 | 4.67 |
|  | Placebo | Sufentanyl PCA | 6 | 9.34 | 1 | 6 | 9.34 |
| Midathala et al (2022) (36) | OL | Tramadol | 72.14 | 18.32 | 0.1 | 7.21 | 1.83 |
|  | TAP | Tramadol | 138.57 | 25.77 | 0.1 | 13.86 | 2.58 |
| Naaz et al (2021) (37) | QL | Cumulative analgesic | 0.74 | 0.61 | 1 | 0.74 | 0.61 |
|  | TAP | Cumulative analgesic | 1.48 | 0.57 | 1 | 1.48 | 0.57 |
|  | Placebo | Cumulative analgesic | 2.68 | 0.56 | 1 | 2.68 | 0.56 |
| Omara et al (2023) (38) | QL | Morphine IV | 10.00 | 1.80 | 1 | 10.00 | 1.80 |
|  | Combined TAP and IIHN block | Morphine IV | 9.90 | 2.40 | 1 | 9.90 | 2.40 |
| Peng et al (2022) (39) | QL | Morphine IV | 5.47 | 4.91 | 1 | 5.47 | 4.91 |
|  | Placebo | Morphine IV | 6.82 | 5.42 | 1 | 6.82 | 5.42 |
| Yousef et al (2018) (42) | QL | Morphine IV | 10.06 | 3.8 | 1 | 10.06 | 3.8 |
|  | TAP | Morphine IV | 14.46 | 3.4 | 1 | 14.46 | 3.4 |

Abbreviations: QL block – Quadratus Lumborum Block; TAP block – Transversus Abdominis Plane Block; IIN block – Ilioinguinal Nerve Block; OSTAP block – Oblique Subcostal Transversus Abdominis Plane Block; ESP block – Erector Spinae Plane Block.
